# Leaf Ethanol Extract of *Dimocarpus longan Lour.* Ameliorates Type 2 Diabetes Mellitus in Rats by Regulating Metabolic Pathways and Gut Microbiota

**DOI:** 10.1155/bri/3881971

**Published:** 2026-01-16

**Authors:** Chunlian Lu, Piaoxue Zheng, Sisi Chen, Yanli Liang, Yuxin Jiang, Anqi Huo, Jingjing Xie, Jiawen Peng, Yuming Ma, Jiali Wei, Zhidong Ma, Hua Zhu, Jie Liang

**Affiliations:** ^1^ College of Pharmacy, Guangxi University of Chinese Medicine, Nanning, 530200, China, gxtcm.com; ^2^ Guangxi Key Laboratory of Zhuang and Yao Ethnic Medicine, Guangxi University of Chinese Medicine, Nanning, 530200, China, gxtcm.com; ^3^ Collaborative Innovation Center of Zhuang and Yao Ethnic Medicine, Guangxi University of Chinese Medicine, Nanning, 530200, China, gxtcm.com; ^4^ Guangxi Zhuang Autonomous Region Ethnic Medicine Resources and Application Engineering Research Center, Guangxi University of Chinese Medicine, Nanning, 530200, China, gxtcm.com; ^5^ Education Department of Guangxi Zhuang Autonomous Region, Key Laboratory of TCM Extraction and Purification and Quality Analysis (Guangxi University of Chinese Medicine), Nanning, 530200, China

**Keywords:** *Dimocarpus longan Lour.* leaves, ethanol extract, gut microbiota, metabolomics, type 2 diabetes mellitus

## Abstract

**Background:**

The leaves of *Dimocarpus longan Lour*. are used unilaterally as Chinese herbal medicines to treat diabetes in Chongzuo and Hezhou, Guangxi, but the mechanism of its treatment of diabetes is not yet clear, and further research is needed.

**Methods:**

This study examined the effects of leaf ethanol extract of *D.s longan Lour.* on metabolic pathways and gut microbiota in rats with type 2 diabetes mellitus (T2DM). The rats were randomly divided into four groups: HG + HFD (T2DM model, fed with high‐sugar and high‐fat diet), control (regular diet), MET (positive metformin), and LYY (leaf ethanol extract of *D. longan Lour*). Metabolite profiles and gut microbiota composition were analyzed using liquid chromatography_mass spectrometry and 16S rDNA sequencing.

**Results:**

Metabolomics analysis revealed 61 distinct metabolites in the LYY group, such as Leu‐Pro and taurolithocholic acid 3‐sulfate, which influence valine, leucine, and isoleucine metabolism, unsaturated fatty acid biosynthesis, fatty acid metabolism, bile secretion, and pyruvate and propanoate metabolism. Additionally, 16S rDNA sequencing showed that LYY significantly altered the abundance of gut microbiota such as *Ligilactobacillus* and *Desulfobacterota* (vs. HG + HFD group, *p* < 0.05).

**Conclusion:**

LYY improved T2DM in rats may be associated with modulating metabolite levels and indirectly regulating glucose metabolism balance through changes in gut microbiota abundance. The efficacy of LYY in treating T2DM in rats may be related to the regulation of six metabolic pathways; it increased the abundance of *Ligilactobacillus* and *Christensenellaceae _ R-7 _ group* and decreased the abundance of *Desulfobacterota*, *Colidextribacter*, and *Oscillibacter*, thereby promoting impaired glucose tolerance and indirectly regulating the balance of glucose metabolism.

## 1. Introduction

Type 2 diabetes mellitus (T2DM) is a systemic metabolic disease and the most prevalent form of clinical diabetes, accounting for 90% of cases worldwide [1]. The incidence of T2DM is continuously increasing due to genetic, environmental, and lifestyle factors, posing a major global health challenge. Metabolites play a critical role in the development of T2DM. For instance, fatty acid, bile acid, and histidine are crucial for regulating glycolipid metabolism and enhancing insulin sensitivity [2, 3]. Notably, glyceride and acylcarnitine lipid metabolites are positively correlated with T2DM [4]. Studies conducted globally have consistently shown that gut microbiota are closely related to T2DM. For instance, Li et al. revealed that administering Bifidobacterium lactis to rats with T2DM significantly improved their condition by regulating gut microbiota composition, fasting blood glucose (FBG) levels, impaired glucose tolerance, and insulin resistance [5]. Gurung showed that Bifidobacterium, Bacteroides, Fusobacterium, Akkermansia, and Roseobacter are negatively correlated with T2DM, whereas Ruminococcus, Fusobacterium, and Cyanobacteria are positively correlated with T2DM [6]. Therefore, restoring metabolic pathways and gut microbiota balance has become a therapeutic goal in managing T2DM.

As a traditional Chinese medicine used to treat diabetes, *Dimocarpus longan Lour.* leaves are known for their hypoglycemic effects. Our research group previously demonstrated that the 95% ethanol extract of *D. longan Lour.* leaves can reduce the FBG levels of different diabetic animal models to varying degrees, showing certain hypoglycemic activity, but has no significant effect on normal FBG [7]. The UPLC‐Q‐Orbitrap HRMS technique identified 9 compounds in *D. longan Lour.* leaves, including luteolin, kaempferol, quercetin, flavonoids and flavonoid glycosides of luteolin, organic acid compounds of shikimic acid and citric acid, and nitrogen‐containing substances of L‐tyrosine, adenosine, and nicotinamide [8, 9]. The main constituents and concentrations of *D. longan Lour.* leaves are summarized in Table 1. The ethyl acetate extract of *D. longan Lour*. leaves has a hypoglycemic effect on type 2 diabetic rats induced by a high‐sugar and high‐fat diet combined with STZ [10]. And in vitro studies have shown that the leaf components inhibit α‐glucosidase activity [11, 12]. In addition, we have also conducted preliminary research on the metabolites in rats of the ethanol extract of *D. longan Lour*. leaves, and the results showed that the metabolites of quercetin, luteolin, and kaempferol were identified in rat fecal samples [13]. And we also have studied the quality control (QC) of *D. longan Lour.* leaves before [14]. However, these research results only indicate that the *D. longan Lour.* leaf extract has a hypoglycemic effect and identify the metabolites of *D. longan Lour.* leaves in rat feces, but do not conduct in‐depth research on the mechanism by which *D. longan Lour.* leaves treat T2DM through regulating metabolism; the mechanism remains unclear and requires further investigation. Therefore, this study conducts an in‐depth investigation based on previous research: employs nontargeted metabolomics and 16S rDNA sequencing to investigate the effects of the leaf ethanol extract of *D. longan Lour.* (LYY) on metabolites and gut microbiota in rats with T2DM, integrates and analyzes the correlation between metabolomics and gut microbiota, conducts a combined analysis on how the ethanol extract of *D. longan Lour*. leaves exerts its therapeutic effect on T2DM by regulating metabolism and gut microbiota, and clarifies its mechanism of action. Moreover, this study provided scientific evidence for the use of *D. longan Lour.* leaves in T2DM treatment.

**Table 1:** Summary table of the main constituents and concentrations of ethanol extract of *D. longan Lour.* leaves.

Title	Molecular formula	Category	Concentrations (mg/g)
Quercetin [8]	C_21_H_20_O_11_	Flavonoid	1.7950 [14]
Luteolin [8]	C_15_H_10_O_6_	Flavonoid	0.0206 [14]
Kaempferol [8]	C_15_H_10_O_6_	Flavonoid	0.4168 [14]
Gallic acid [9]	C_7_H_6_O_5_	Phenolic acids	1.3077 [14]
Catechuic acid [9]	C_7_H_6_O_4_	Phenolic acids	0.1214 [14]
Ethyl gallate [8]	C_9_H_10_O_5_	Phenolic acids	0.1338 [14]

## 2. Materials

### 2.1. Laboratory Animal

Thirty healthy Sprague–Dawley rats (200 ± 20 g, male, grade SPF) were purchased from Hunan Slack Jingda Experimental Animal Co., Ltd. [license number: SCXK (Xiang) 2021‐0004]. This study was approved by the Ethics Committee of Guangxi University of Chinese Medicine (approval number: DW20230102‐028).

### 2.2. Reagents and Instruments

The leaves of *D. longan Lour.* were collected from Hezhou, Guangxi, and identified as belonging to the family Sapindaceae by Professor Teng Jianbei (School of Pharmacy, Guangxi University of Traditional Chinese Medicine). Rat maintenance feed was provided by Beijing Keao Co., Ltd. (China), and high‐sugar and high‐fat feed were obtained from Beijing Bo’ai Port Business Center (China). Metformin hydrochloride was supplied by Beijing Jingfeng Pharmaceutical Group Co., Ltd. (China), and streptozotocin (STZ) was purchased from Sigma Corporation (USA). Total cholesterol (TC), triglyceride (TG), low‐density lipoprotein cholesterol (LDL‐C), and high‐density lipoprotein cholesterol (HDL‐C) kits were procured from Nanjing Jiancheng Bioengineering Research Institute (China). Anhydrous ethanol and sodium pentobarbital were obtained from Chengdu Kelong Chemical Co., Ltd. (China), whereas methanol, formic acid, and ammonium acetate were purchased from Thermo Fisher Corporation (USA). Water was obtained from Merck (Germany). Magnetic bead method soil, fecal genomic DNA extraction kit, and Universal DNA Purification and Recovery Kit were obtained from TianGen China Company, whereas Phusion^R^ High‐Fidelity PCR Master Mix with GC Buffer, Phusion^R^ High‐Fidelity DNA Polymerase Enzyme, and NEB Next^R^ Ultra ll FS DNA PCR‐free Library Prep Kit were purchased from New England Biolabs (USA).

The following laboratory equipment was used: SQP analysis balance (Sartorius, Germany), electronic balance (0.1–1000 g; Zhejiang Kaifeng Group Company, China), HH‐4 digital display constant temperature water bath (Changzhou Guohua Electric Co., Ltd., China), OSB‐2200 rotary steam meter (Eyela, Japan), H1650‐W centrifuge (Xiangyi Centrifuge Instrument Co., Ltd., China), Anwen BGMS‐1 blood glucose meter and blood glucose test strip (Changsha Sinocare Biosensing Technology Co., Ltd., China), full‐wavelength Multiskan Sky microplate reader (Thermo Scientific, USA), Q Exactive HF/Q Exactive HF‐X mass spectrometer and Vanquish UHPLC chromatograph (Thermo Fisher, Germany), D3024R cryogenic centrifuge (Scilogex, USA), Hypesil Gold column (100 × 2.1 mm, 1.9 μm; Thermo Fisher, USA), CTAB extractor (Nobleryder China Company), T100 gradient PCR instrument (Bio‐Rad), and NovaSeq 6000 tester (lllumina, San Diego, CA, USA).

## 3. Methods

### 3.1. Extraction

Crude powder (1000 g) from *D. longan Lour.* leaves was extracted 6 times using 95%, 75%, and 50% ethanol solutions. The mixtures were heated and refluxed thrice for 2 h each time. The filtrates were mixed, and ethanol was recovered. The extract was concentrated and dried until no alcohol odor remained, yielding a leaf ethanol extract (extract rate: ∼30%), which was stored at 4°C for subsequent use [13].

### 3.2. Animal Modeling

After 1 week of adaptive feeding, the rats were randomly categorized into the normal diet group or high‐glucose, high‐fat diet group. After 12 h, of fasting, the high‐glucose, high‐fat diet group received an intraperitoneal injection of streptomycin at a low dose (40 mg kg^−1^) and continued on the diet for 4 weeks. Then, all rats were fasted for 12 h, and the FBG level in the high‐glucose, high‐fat diet group was measured via tail blood sampling, with values exceeding 16.7 mmol L^−1^. Rats in this group also exhibited polydipsia, polyphagia, polyuria, and weight loss, indicating successful establishment of the T2DM model [15].

### 3.3. Animals and Experimental Design

The established rat models were randomized into four groups, with six rats per group: T2DM model, fed with high‐sugar and high‐fat diet (HG + HFD) group, regular diet (control) group, positive metformin (MET) group, and leaf ethanol extract of *D. longan Lour*. (LYY) group. Rats in the LYY group received LYY (5.34 g·kg^−1^·day^−1^), whereas those in the positive MET group received metformin hydrochloride (100 mg·kg^−1^·day^−1^). Pure water at the same volume was administered to the control and HG + HFD groups. Rat hair color and mental state were assessed daily, and their body weight and FBG levels were recorded weekly. The experimental period lasted 4 weeks.

### 3.4. Blood Glucose and Lipid Tests

In the intervention and control groups, rats were fasted for 12 h weekly before FBG was measured via tail blood sampling. After 4 weeks, the rats underwent 12‐h fasting before the oral glucose tolerance test (OGTT). FBG level was measured in tail blood, and 40% glucose solution was administered at a dose of 5 mL kg^−1^. Blood glucose was measured at 30, 60, 90, and 120 min. An OGTT curve was plotted using the formula shown below. Rats were anesthetized via intraperitoneal injection of 5% sodium pentobarbital (purity > 99.5%), and blood was collected from the abdominal aorta. The blood was centrifuged at 3000 rpm for 10 min, and TC, TG, LDL‐C, and HDL‐C were measured from the upper layer of transparent serum.

The area under the curve (AUC) was calculated using the following formula:

(1) AUC=A/2+B+C+D+E/22,

where *A*, *B*, *C*, *D*, and *E* indicate the 0‐, 30‐, 60‐, 90‐, and 120‐min blood glucose levels, respectively.

### 3.5. Fecal Untargeted Metabolomics Assays

A 100‐mg fecal sample was collected and immersed in an EP tube containing 80% aqueous methanol solution. The sample was vortexed for 60 s, rested on an ice bath for 5 min to precipitate proteins, and centrifuged at 15, 000 × *g* and 4°C for 20 min. The supernatant was diluted to the level of 53% methanol using mass spectrometry (MS) water, centrifuged again at 15, 000 × *g* and 4°C for 20 min, and subjected to liquid chromatography (LC)–MS analysis [16]. Separation was conducted using Vanquish UHPLC chromatography and a Hypersil GOLD column, with Q Exactive HF/Q Exactive HF‐X MS employed for analysis. The primary and secondary spectra of QC samples were obtained for metabolite identification. Linux OS CentOS Version 6.6 was used to preprocess the data. Metabolites were annotated in the Kyoto Encyclopedia of Genes and Genomes (KEGG), Human Metabolome Database (HMDB), and LIPID MAPS databases. Metabolomics data were analyzed via multivariate statistical methods, and volcano plots were constructed using R software.

### 3.6. 16S rDNA Amplicon Sequencing of Gut Microbiota

Fecal DNA was extracted using a fecal genomic DNA extraction kit. The samples were centrifuged and diluted to 1 ng·μL^−1^ with sterile water. Its purity and concentration were determined via 1% agarose gel electrophoresis.

Polymerase chain reaction (PCR) was used to amplify the V4 variable region using Barcode and high‐fidelity DNA polymerase. The primers used, in order, were 515F and 806R. PCR amplification was confirmed through 2% agarose gel electrophoresis, and purified PCR products were obtained using magnetic beads [17]. Library construction was performed using NEB Next ⟶ Ultra II FS DNA PCR‐free Library Prep Kit. After achieving satisfactory library quality, PE 250 on‐machine sequencing was conducted using NovaSeq 6000.

Paired‐end sequences were spliced using FLASH to generate raw data, and fastp Version 0.23.1 was used to process and filter the original data, removing chimeras to yield valid data [18, 19]. Operational taxonomic unit (OTU) clustering was performed using UPGMA, with species annotation achieved using QIIME2 and the Silva 138.1 annotation database. Analyses of OTU abundance, α‐ and β‐diversity, and principal components as well as statistical analysis methods, such as MetaStat, were conducted to examine significant intergroup differences in species composition.

### 3.7. Combined Correlation Analysis of Metabolomics and Gut Microbiota

Spearman correlation analysis was conducted to examine the relationship between the significantly different metabolites identified via metabolomics analysis and the significantly different gut microbiota at the genus level detected via 16S rDNA sequencing.

### 3.8. Data Analysis

Statistical analyses were performed using GraphPad Prism 9.5.0. Data are expressed as means ± standard deviations. Group comparisons were conducted using one‐way analysis of variance (ANOVA), and differences were tested using Student’s *t*‐test. Ordinary ANOVA was used for homogeneous variances, whereas the Brown–Forsythe and Welch ANOVA tests were used for nonhomogeneous variances. Tamhane’s T2 test was employed for multiple comparison corrections. *p*‐values of < 0.05 were considered to indicate statistical significance.

## 4. Results

### 4.1. Impact of LYY on Weight, FBG Levels, and Blood Lipid Content in Rats With T2DM

#### 4.1.1. Effects of LYY on Rat Weight

As shown in Figure 1(a), there were no significant weight differences across groups after 1 week of adaptive feeding before modeling. After treatment intervention (6–9 weeks), weight changes became apparent across all groups. The weight of rats in the LYY group increased significantly compared with that in the HG + HFD group. Following 1 week of treatment intervention, the rat weight in the HG + HFD group decreased significantly compared with that in the control group (*p* < 0.05), showing typical wasting symptoms. Compared with the HG + HFD group, the LYY and MET groups exhibited significant weight gain (*p* < 0.05). After 2–4 weeks of treatment intervention, the LYY and MET groups showed significant weight gain (*p* < 0.01), approaching normal levels. These results indicate that LYY can effectively alleviate physical wasting symptoms in rats in the HG + HFD group.

Figure 1Effects of LYY on (a) weight (weekly measurements), (b) FBG, fasting blood glucose (measurements after 12 h of weekly fasting), (c) OGTT, oral glucose tolerance test (after 12 h of fasting, 40% glucose solution was administered at a volume of 5 mL kg^−1^, and blood glucose values were measured at 5 time points), (d) AUC, calculate the area under the curve (according to the formula in Section 3.4) in different rat groups. Statistical significance by one‐way ANOVA with Tamhane’s T2 test was employed for multiple comparison corrections. Control (regular diet), HG + HFD (T2DM model, fed with high‐sugar and high‐fat diet), MET (positive metformin), and LYY (leaf ethanol extract of *D. longan Lour*.). Number of rats in each group = 6. Number of rats in each group = 6. ^∗∗^ *p* < 0.01 vs. control group; ^##^ *p* < 0.01 vs. HG + HFD group.

**Figure:**
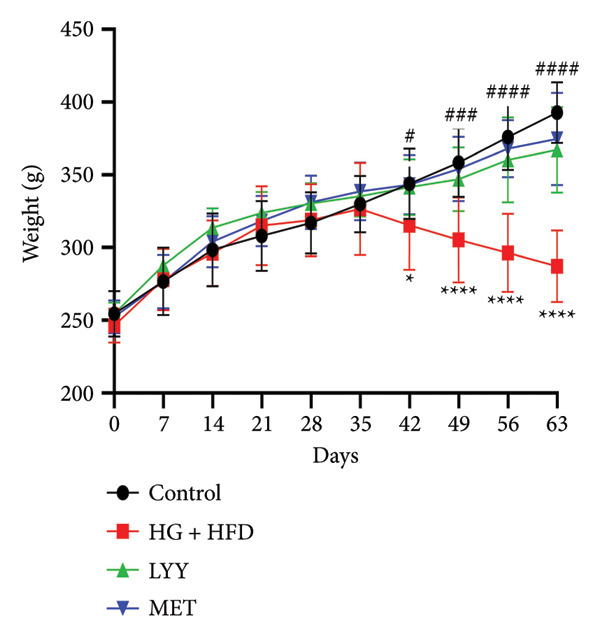
(a)

**Figure:**
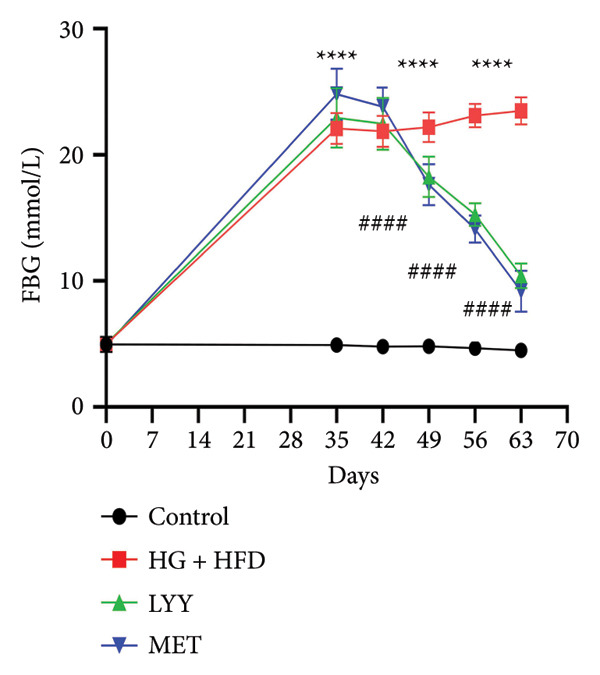
(b)

**Figure:**
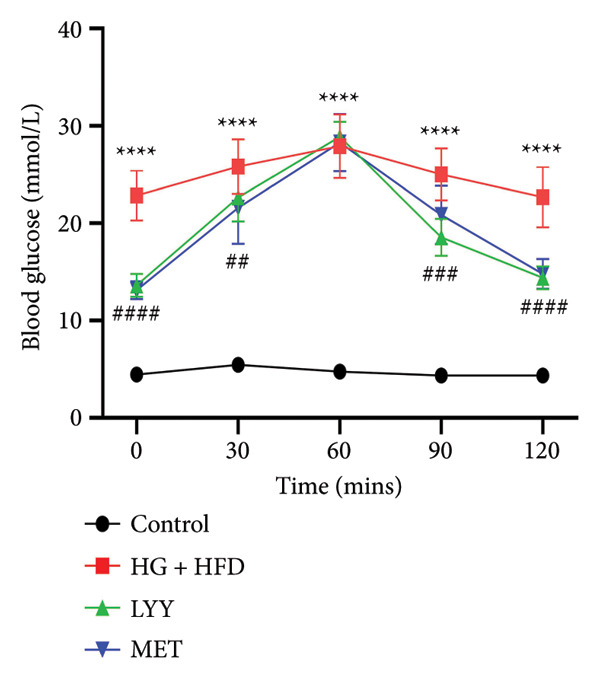
(c)

**Figure:**
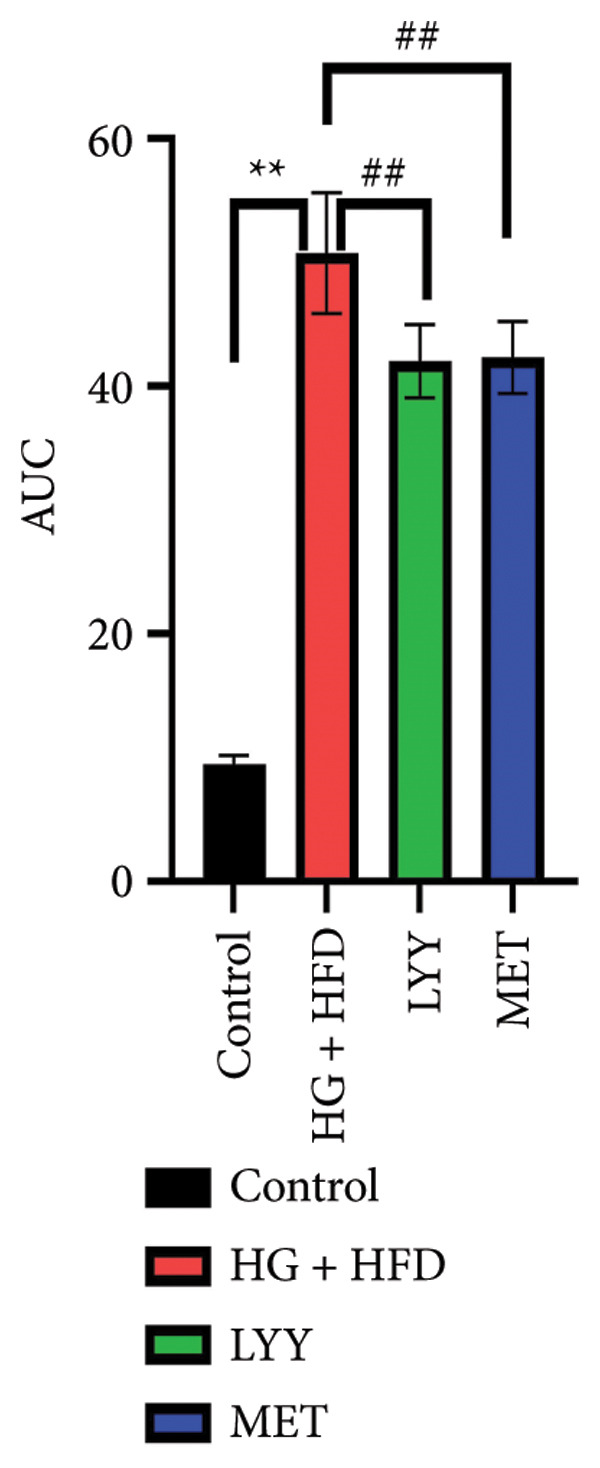
(d)

#### 4.1.2. Effects of LYY on FBG

As shown in Figure 1(b), baseline FBG levels were normal and comparable across all groups before the model was established. Compared with the control group, FBG levels in the HG + HFD, LYY, and MET groups significantly increased (*p* < 0.01), confirming successful establishment of the T2DM rat model. After 2–4 weeks of treatment intervention, FBG levels in the LYY and MET groups significantly decreased compared with those in the HG + HFD group (*p* < 0.01). These findings suggest that LYY effectively regulates abnormal blood glucose levels in rats with T2DM.

#### 4.1.3. Effects of LYY on OGTT Results

As shown in Figure 1(c), the blood glucose levels of rats in the HG + HFD, LYY, and MET groups increased significantly after the administration of glucose solution (*p* < 0.01), with the levels peaking at 60 min before declining and finally reaching approximately the initial value at 120 min. This pattern indicates impaired glucose tolerance in rats with T2DM. As shown in Figure 1(d), the AUC for blood glucose changes was significantly higher in the HG + HFD group than in the control group (*p* < 0.01); however, AUC values for blood sugar changes in the LYY and MET groups were significantly lower than those in the HG + HFD group (*p* < 0.01), suggesting that LYY helps maintain glucose tolerance.

#### 4.1.4. Effects of LYY on Lipid Levels

As shown in Figure 2, compared with the control group, statistically significant reductions in TC (Figure 2(a)), TG (Figure 2(b)), and LDL‐C (Figure 2(c)) levels and a significant increase in the HDL‐C (Figure 2(d)) level were observed in the HG + HFD group (*p* < 0.01). Compared with the HG + HFD group, significant reductions in TG (*p* < 0.05), TC (*p* < 0.01), and LDL‐C (*p* < 0.01) levels were observed in the LYY group, whereas HDL‐C levels were significantly elevated in the LYY group (*p* < 0.05). The MET group displayed significantly reduced TC, TG, and LDL‐C levels and significantly higher HDL‐C levels compared with the HG + HFD group (*p* < 0.01). These results indicate that LYY effectively improves lipid metabolism disorders in rats with T2DM.

Figure 2Effects of LYY on (a) TC, total cholesterol), (b) TG, triglyceride, (c) LDL‐C, low‐density lipoprotein cholesterol, (d) HDL‐C in different rat groups, high‐density lipoprotein cholesterol (according to the method of each kit, the corresponding lipid values were tested and calculated). Statistical significance by one‐way ANOVA with Tamhane’s T2 test was employed for multiple comparison corrections. Control (regular diet), HG + HFD (T2DM model, fed with high‐sugar and high‐fat diet), MET (positive metformin), and LYY (leaf ethanol extract of *D. longan Lour*.). Number of rats in each group = 6. ^∗∗^ *p* < 0.01 vs. control group; ^#^ *p* < 0.05, ^##^ *p* < 0.01 vs. HG + HFD group.

**Figure:**
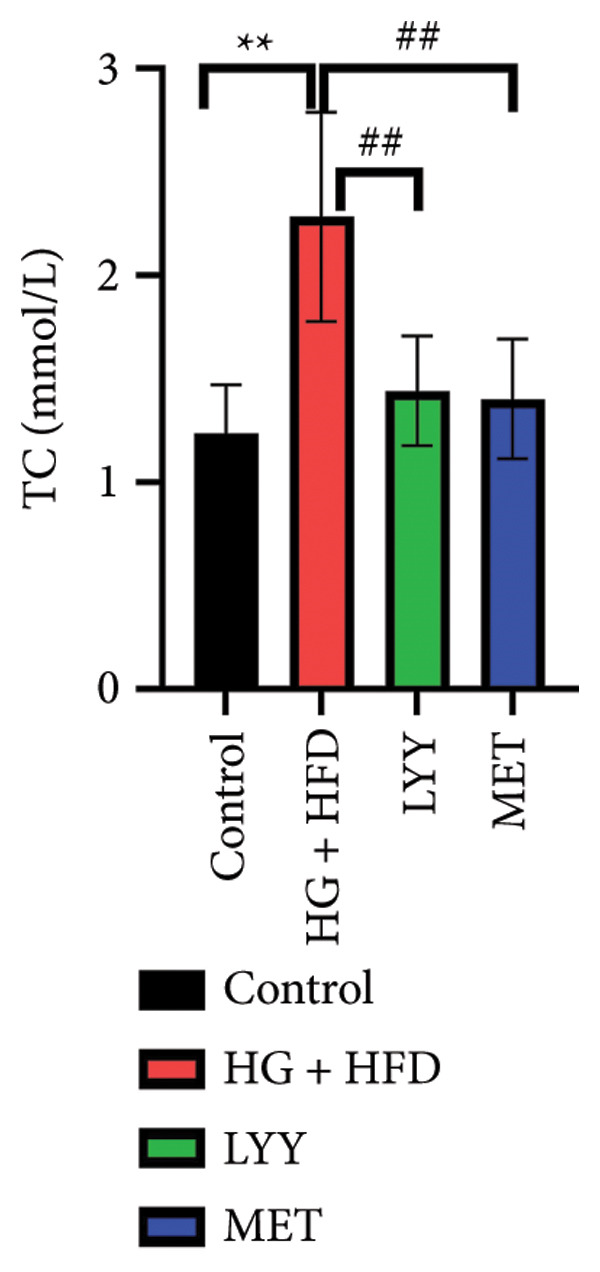
(a)

**Figure:**
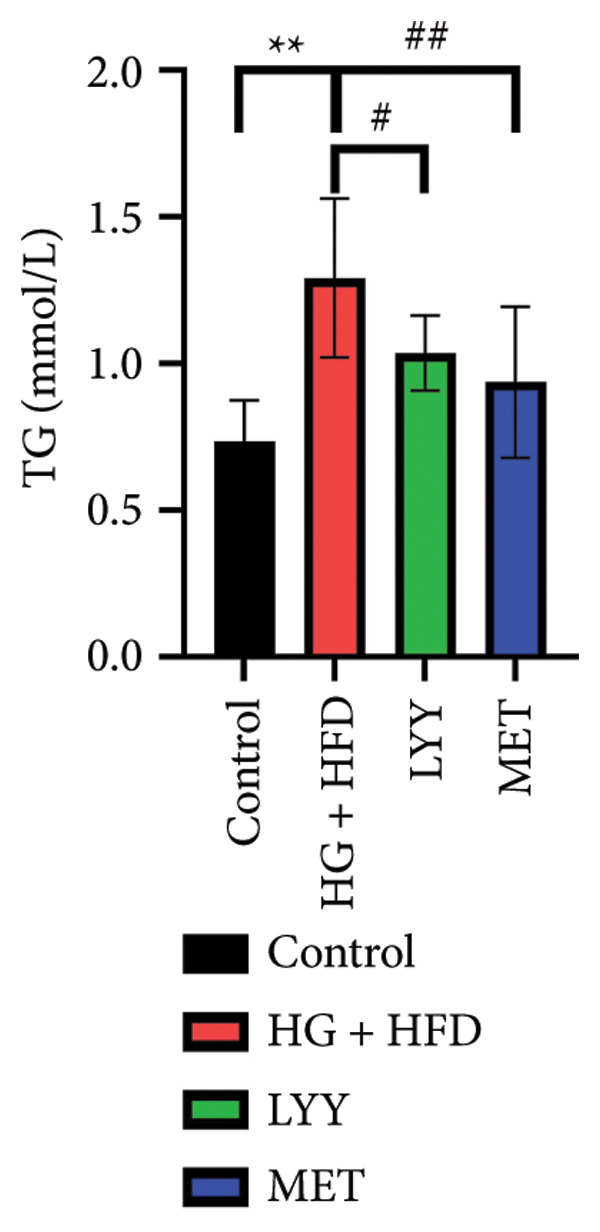
(b)

**Figure:**
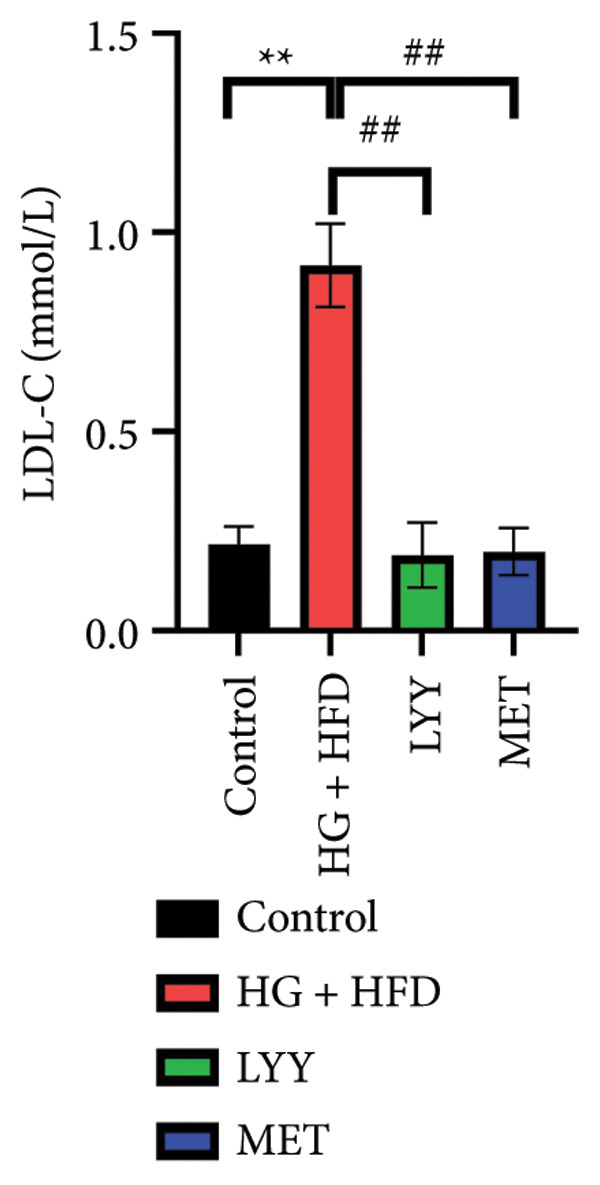
(c)

**Figure:**
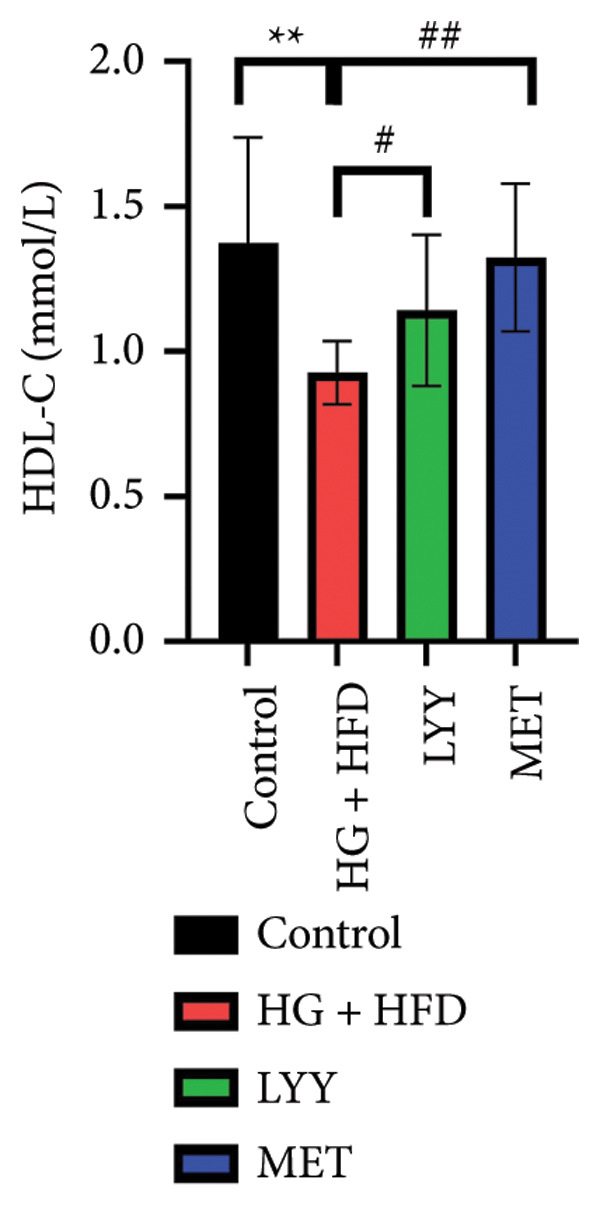
(d)

### 4.2. Impact of LYY on Fecal Metabolomics in Rats With T2DM

#### 4.2.1. QC Results From LC–MS

QC sample correlation analysis indicated that the QC samples had strong correlation (*R* = 0.99–1.00) in positive and negative ion modes, confirming stability throughout the detection process. Principal component analysis showed that the QC samples clustered in positive and negative ion modes, with denser and smaller distributions, reflecting high method stability and data quality.

#### 4.2.2. Differential Metabolite Identification Analysis

As shown in Figure 3, comparative analysis of samples in positive and negative ion modes using the partial least squares discriminant analysis (PLS‐DA) model revealed significant differences between the HG + HFD and control, LYY, and MET groups. Pairwise comparisons in both modes showed clear differences between the control, HG + HFD, LYY, and MET groups, indicating that the T2DM model and subsequent treatment intervention caused significant changes in gut metabolite concentrations in rats. To analyze these metabolite changes, a volcano plot (Figure 4) was constructed based on VIP scores, fold change (FC) values, and *t*‐test results, with red and green dots representing significantly upregulated and downregulated metabolites, respectively. As shown in Figure 4, in positive and negative ion models, HG + HFD vs. control (Figures 4(a) and 4(b)), LYY vs. HG + HFD (Figures 4(c) and 4(d)), and MET vs. HG + HFD (Figures 4(e) and 4(f)) groups showed significant differences in the up‐ or downregulation of positive and negative ion metabolites. These metabolites that differed significantly between the HG + HFD and control groups were screened according to the following criteria: FC > 1.2 or < 0.833, and *p* < 0.05, in the PLS‐DA model VIP > 1.0. The screened metabolites were classified and analyzed in the LYY group using KEGG, HMDB, and LIPID MAPS databases. As shown in Table 2, 61 common metabolites were potentially identified between the HG + HFD and control groups and between the LYY and HG + HFD groups. Following LYY treatment intervention, lipids, such as 2‐methylbutyroylcarnitine, (2‐aminoethoxy) [(2R)‐3‐hydroxy‐2‐(pentadecanoyloxy) propoxy] phosphonic acid] (LPE 15:0), and 1‐tridecanoyl‐sn‐glycero‐3‐phosphate serine (LPS 13:0) as well as fatty acids, such as 5‐oxo‐6E, 8Z, 11Z, 14Z‐eicosadetraenoic acid (5‐OxoETE), and ureidosuccinic acid, were significantly upregulated. Other lipids, such as O‐octadecanoyl‐R‐carnitine (CAR 18:0) and 1‐(9Z‐octadecenoyl)‐sn‐glycero‐3‐phosphate‐(1′‐sn‐glycerol) (LPG 18:1); fatty acids, such as 17S‐hydroxy‐5Z, 8Z, 11Z, and 14Z‐eicosadetraenoic acid [17 (S)‐HETE]; and amino acids, such as Leu‐Pro, (S)‐leucic acid, taurolithocholic acid 3‐sulfate, histamine, medroxyprogesterone, and 6β‐hydroxytestosterone, were significantly downregulated. These results suggest that LYY can alleviate T2DM symptoms associated with regulating fecal metabolites.

Figure 3Partial least squares discriminant analysis (PLS‐DA) score chart of positive and negative ion models. The abscissa is the score of the sample on the first principal component; the ordinate is the score of the sample on the second principal component; R2Y represents the interpretation rate of the model, Q2Y is used to evaluate the predictive ability of the PLS‐DA model, and when R2Y is greater than Q2Y, it means that the model is well established. Control (regular diet), HG + HFD (T2DM model, fed with high‐sugar and high‐fat diet), MET (positive metformin), and LYY (leaf ethanol extract of *D. longan Lour*.). Number of rats in each group = 6. (a) PLS‐DA of positive ion models. Control vs. HG + HFD. (b) PLS‐DA of negative ion models. Control vs. HG + HFD. (c) PLS‐DA of positive ion models. LYY vs. HG + HFD. (d) PLS‐DA of negative ion models. LYY vs. HG + HFD. (e) PLS‐DA of positive ion models. HG + HFD vs. MET. (f) PLS‐DA of negative ion models. HG + HFD vs. MET.

**Figure:**
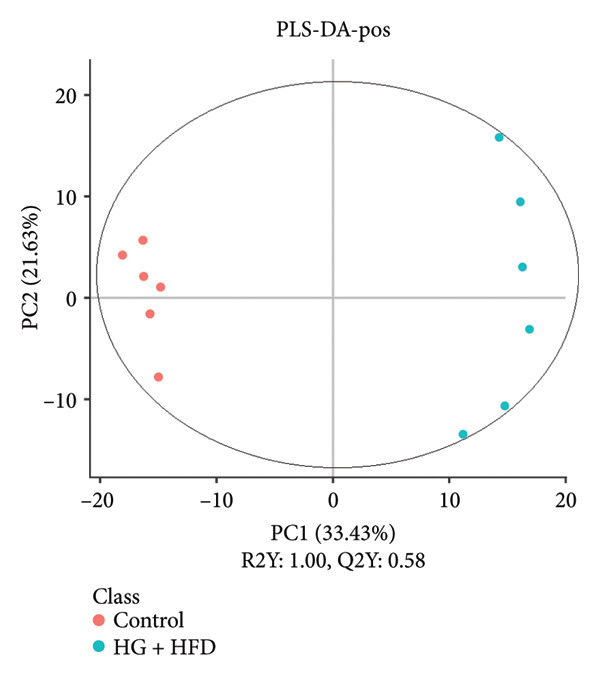
(a)

**Figure:**
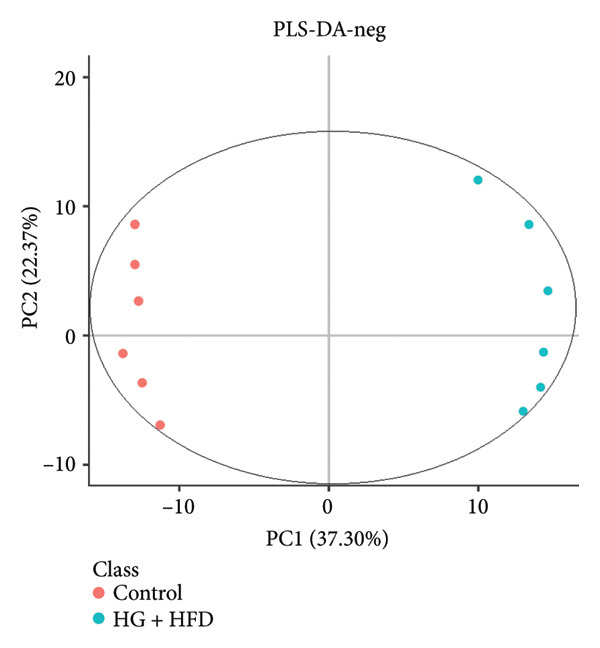
(b)

**Figure:**
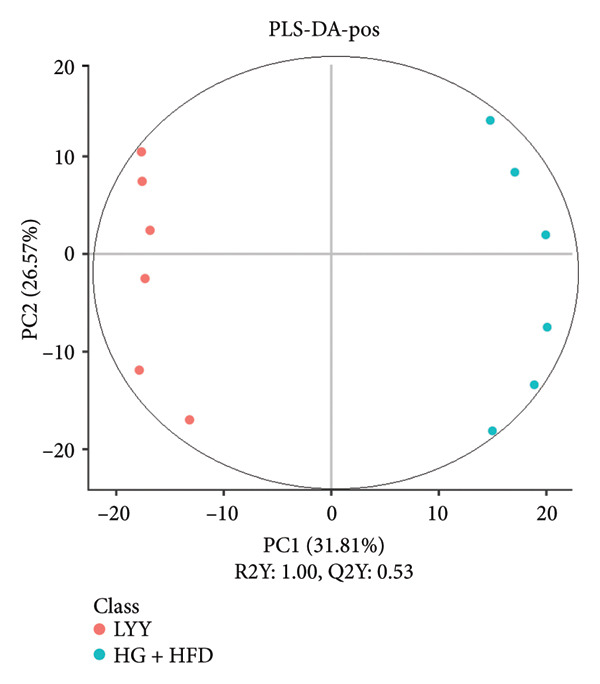
(c)

**Figure:**
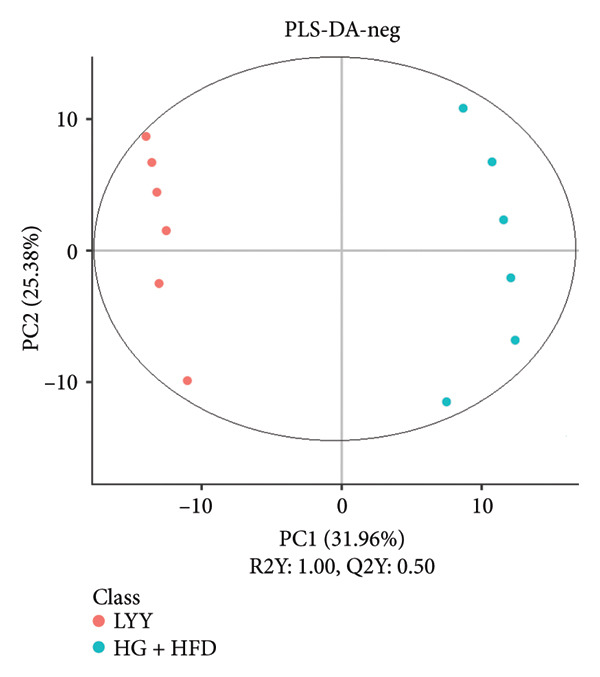
(d)

**Figure:**
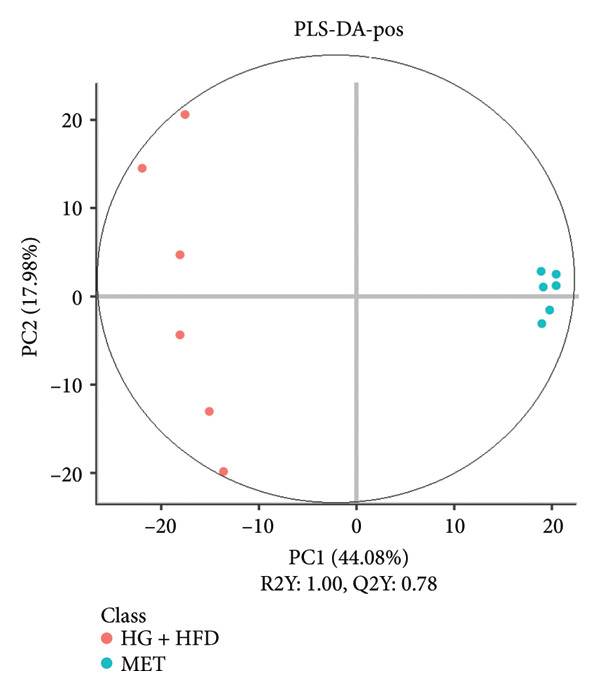
(e)

**Figure:**
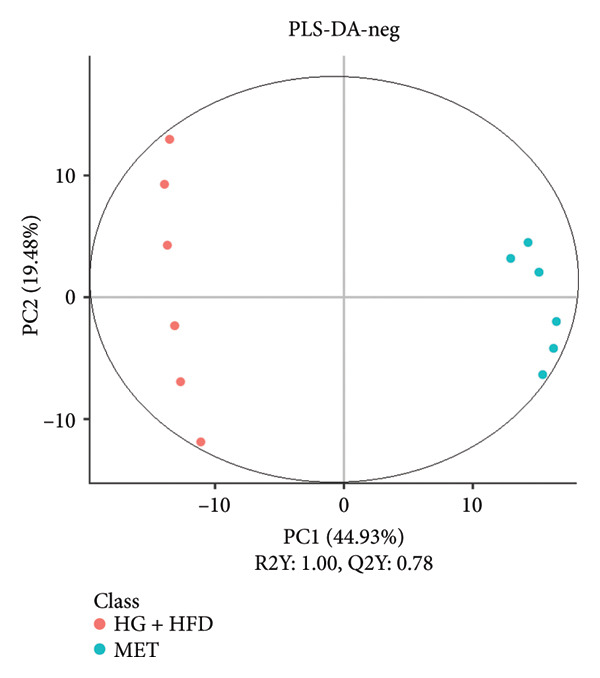
(f)

Figure 4Volcanic map of positive and negative ion models. HG + HFD vs. control: (a) positive, (b) negative; LYY vs. HG + HFD: (c) positive, (d) negative; MET vs. HG + HFD: (e) positive, (f) negative. VIP > 1.0, metabolites significantly elevated on the volcano plot were represented by red dots, and metabolites significantly reduced were represented by green dots. Control (regular diet), HG + HFD (T2DM model, fed with high‐sugar and high‐fat diet), MET (positive metformin), and LYY (leaf ethanol extract of *D. longan Lour*.).

**Figure:**
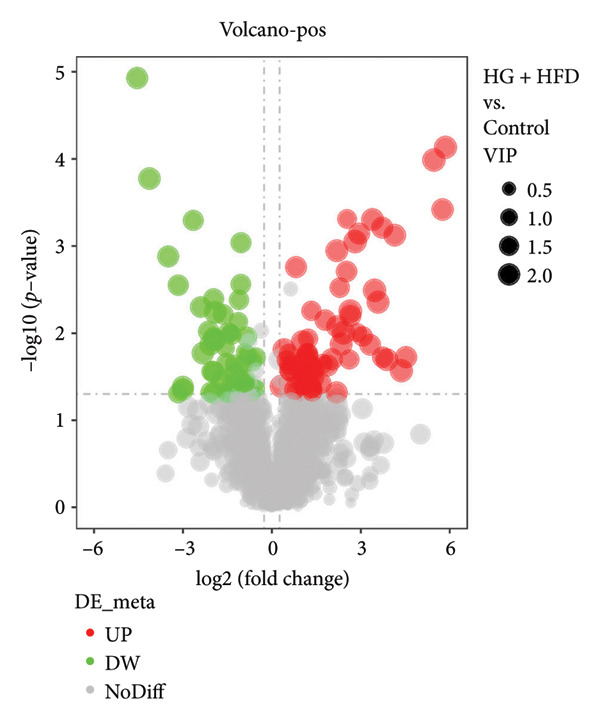
(a)

**Figure:**
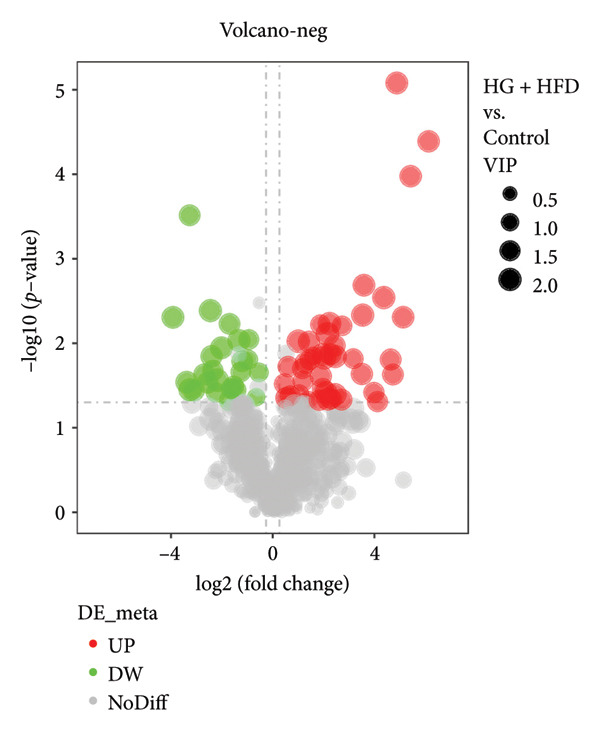
(b)

**Figure:**
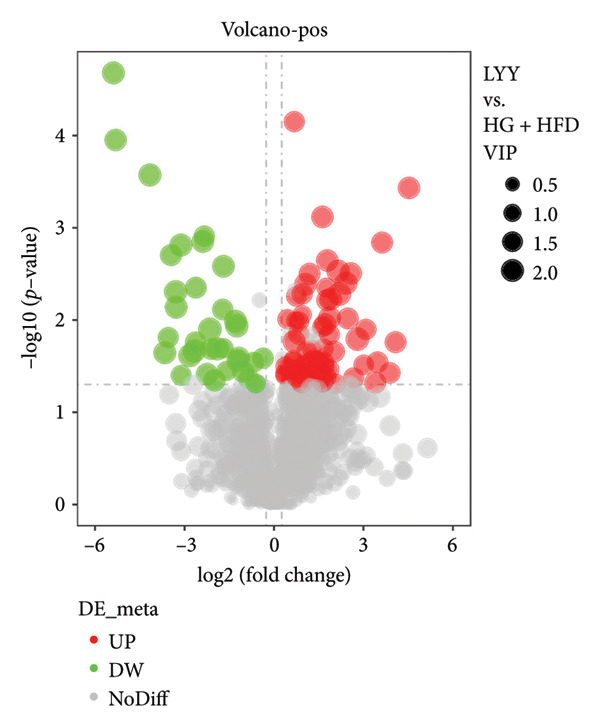
(c)

**Figure:**
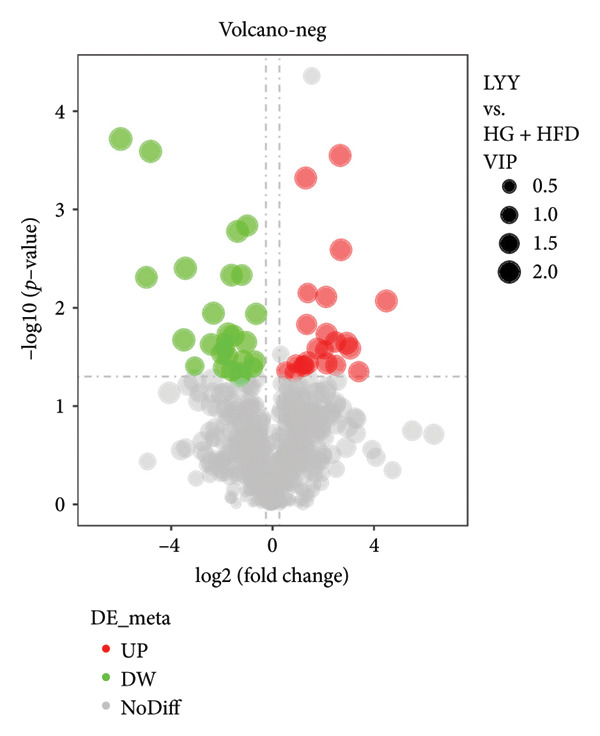
(d)

**Figure:**
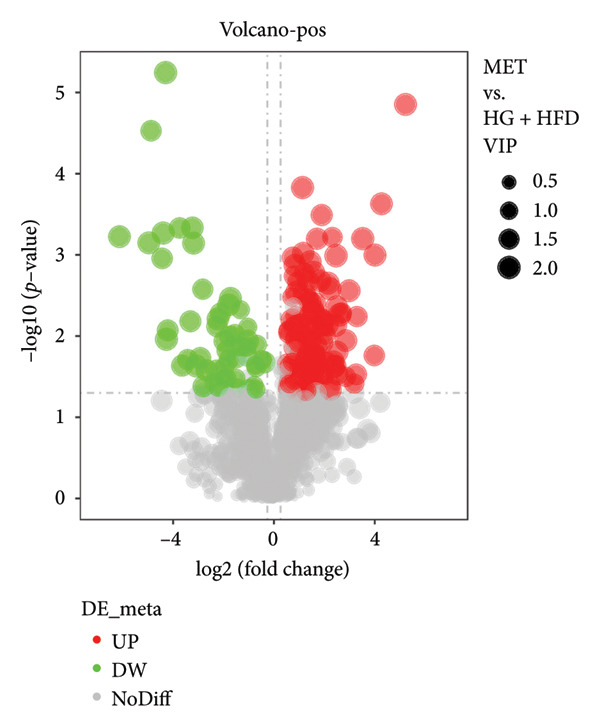
(e)

**Figure:**
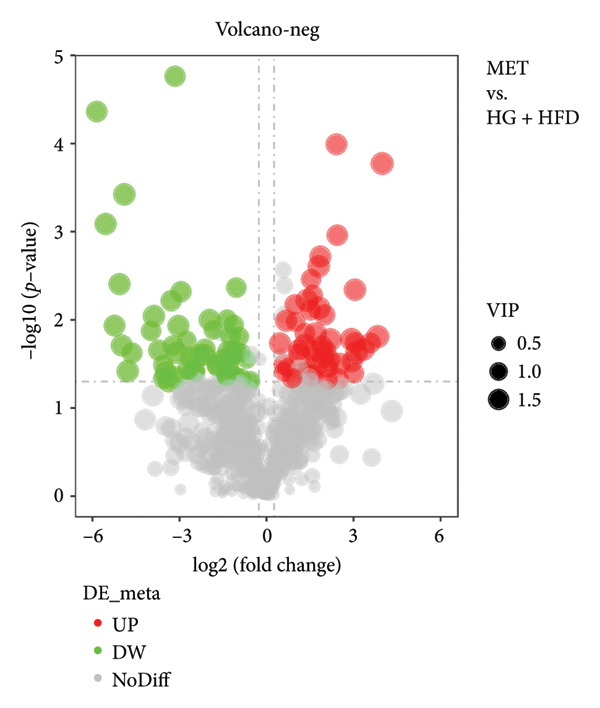
(f)

**Table 2:** Potential differential metabolite information in rat feces.

Number	Title	HMDB ID	FC	*p* value	VIP	Up/down	
						HG + HFD vs. control	LYY vs. HG + HFD
1	Noroxycodone‐d3	—	5.34	0.009	2.40	↑	↓
2	Histamine	0,000,870	44.23	0.0001	2.36	↑	↓
3	C‐6 NBD ceramide	—	11.01	0.003	2.34	↑	↓
4	VMK	—	6.99	0.0008	2.34	↑	↓
5	T‐2 Triol	0,035,846	58.00	7.34E‐05	2.29	↑	↓
6	(S)‐leucic acid	0,000,746	11.96	0.004	2.23	↑	↓
7	13,14‐dihydro‐15‐keto‐tetranor prostaglandin E2	—	10.49	0.0004	2.19	↑	↓
8	Leu‐Pro	0,011,175	17.70	0.0007	2.15	↑	↓
9	Norverapamil	0,060,540	14.71	0.02	2.15	↑	↓
10	Taurolithocholic acid 3‐sulfate	0,002,580	70.16	8.3E‐06	2.14	↑	↓
11	4‐[2‐(5‐bromo‐2‐thienyl)‐4‐pyrimidinyl]benzamide	—	0.057	0.0001	2.14	↓	↑
12	Mupirocin	0,014,554	54.16	0.0003	2.12	↑	↓
13	3,14‐dihydro‐15‐keto‐tetranor prostaglandin E2	—	35.07	0.0001	2.08	↑	↓
14	5‐fluoro‐2‐[(3S)‐1‐(2‐fluorobenzyl)‐3‐pyrrolidinyl]‐1H‐benzimidazole	—	4.55	0.04	2.06	↑	↓
15	16‐(hexopyranosyloxy)‐7‐hydroxy‐8,9‐epoxypimaran‐18‐oic acid	—	26.53	0.002	2.05	↑	↓
16	LPG 18:1	—	17.41	0.004	2.02	↑	↓
17	Piperine	0,029,377	0.042	1.18E‐05	2.01	↓	↑
18	QNK	—	0.26	0.01	2.00	↓	↑
19	1‐ethyl 4‐(2‐oxo‐1,2‐diphenylethyl) succinate	—	0.088	0.001	1.99	↓	↑
20	7‐ketocholesterol	0,000,501	15.80	0.004	1.98	↑	↓
21	Coenzyme Q2	0,006,709	0.42	0.02	1.98	↓	↑
22	cyclohexyl{4‐[4‐nitro‐2‐(1H‐pyrrol‐1‐yl)phenyl]piperazino}methanone	—	13.19	0.0006	1.97	↑	↓
23	Dextrorphan	0,060,552	7.69	0.0007	1.96	↑	↓
24	6ß‐hydroxytestosterone	—	2.36	0.02	1.96	↑	↓
25	4‐{4‐[3‐(4‐chlorophenoxy)propyl]piperazino}‐1H‐indole	—	6.28	0.006	1.94	↑	↓
26	2‐isopropylmalic acid	0,000,402	7.58	0.02	1.94	↓	↑
27	N,6‐diphenylthieno[2,3‐d]pyrimidin‐4‐amine	—	6.62	0.006	1.93	↑	↓
28	Tramadol N‐oxide	—	0.24	0.01	1.91	↓	↑
29	TMK	—	3.48	0.007	1.86	↑	↓
30	Methanandamide	0,243,685	0.19	0.005	1.85	↓	↑
31	5‐OxoETE	0,010,217	0.23	0.009	1.85	↓	↑
32	CAR 18:0	—	13.34	0.01	1.84	↑	↓
33	Isophorone	0,031,195	0.39	0.03	1.81	↓	↑
34	Thromboxane B3	0,005,099	3.93	0.01	1.80	↑	↓
35	1‐(4‐benzylpiperazino)‐2‐(pyridin‐2‐ylamino)propan‐1‐one	—	0.24	0.02	1.79	↓	↑
36	17alpha‐ethinyl estradiol	0,001,926	0.16	0.0005	1.76	↓	↑
37	3‐(2‐thienyl)‐1,2,4‐oxadiazole‐5‐carbohydrazide	—	0.26	0.004	1.73	↓	↑
38	15(R),19(R)‐hydroxy prostaglandin F1*α*	—	2.64	0.01	1.72	↑	↓
39	2‐butoxyacetic acid	0,041,844	2.26	0.02	1.71	↓	↑
40	Methylmalonate	0,000,202	14.81	0.04	1.70	↓	↑
41	17(S)‐HETE	—	2.03	0.02	1.63	↑	↓
42	1,7‐diphenylhept‐4‐en‐3‐one	0,031,873	0.32	0.006	1.63	↓	↑
43	Medroxyprogesterone	0,001,939	2.52	0.005	1.60	↑	↓
44	Succinic acid	0,000,828	10.54	0.04	1.60	↓	↑
45	Stearic acid	0,000,827	0.16	0.02	1.59	↑	↓
46	Azelaic acid	0,000,784	0.43	0.03	1.54	↓	↑
47	LPE 15:0	—	0.36	0.03	1.53	↓	↑
48	LPS 13:0	—	0.32	0.03	1.52	↓	↑
49	DL‐panthenol	0,304,820	0.35	0.04	1.47	↓	↑
50	(±)9‐HpODE	—	0.25	0.03	1.46	↓	↑
51	Ureidosuccinic acid	0,000,828	0.34	0.02	1.45	↓	↑
52	Diflorasone	0,014,368	0.25	0.01	1.44	↓	↑
53	Artemisinin	—	2.62	0.02	1.44	↑	↓
54	XLR11 N‐(4‐hydroxypentyl) metabolite	—	0.39	0.01	1.42	↓	↑
55	NMK	—	0.42	0.02	1.40	↓	↑
56	2‐methylbutyroylcarnitine	0,000,378	0.46	0.007	1.37	↓	↑
57	Oleic acid	0,000,207	0.65	0.04	1.36	↑	↓
58	P‐mentha‐1,3,8‐triene	0,037,013	0.59	0.04	1.29	↓	↑
59	N‐(2,3‐dihydro‐1,4‐benzodioxin‐6‐yl)‐2,5‐dimethyl‐3‐furamide	—	0.54	0.03	1.26	↓	↑
60	6‐amino‐1‐(2‐methylphenyl)‐1,2,3,4‐tetrahydropyrimidine‐2,4‐dione	—	0.38	0.009	1.12	↓	↑
61	Acetoacetate	0,304,256	1.64	0.01	1.11	↓	↑

#### 4.2.3. Metabolic Pathway Analysis

Differential metabolites were analyzed through KEGG pathway enrichment, with significance level set at *p* < 0.05 (Figure 5). In Figure 5, under the positive ion model, the differential metabolic pathways with a *P*‐value less than 0.05 include valine, leucine, and isoleucine degradation, propanoate metabolism, fatty acid biosynthesis, and biosynthesis of unsaturated fatty acids. Under the negative ion model, the differential metabolic pathways with a *P*‐value less than 0.05 are bile secretion and pyruvate metabolism. In conclusion, key metabolic pathways distinguishing the LYY and HG + HFD groups were as follows: valine, leucine, and isoleucine degradation, biosynthesis of fatty acid and unsaturated fatty acids, bile secretion, pyruvate metabolism, and propanoate metabolism. These pathways suggest that LYY’s effect on improving T2DM in rats is closely related to these metabolic pathways.

Figure 5Analysis of KEGG pathway enrichment of differential metabolite between LYY group and HG + HFD group under positive and negative ion models: (a) positive; (b) negative. The size of the dots represents the number of differential metabolites in the corresponding pathway, and the larger it is, the more differential metabolites are within the pathway. HG + HFD (T2DM model, fed with high‐sugar and high‐fat diet), and LYY (leaf ethanol extract of *D. longan Lour*.).

**Figure:**
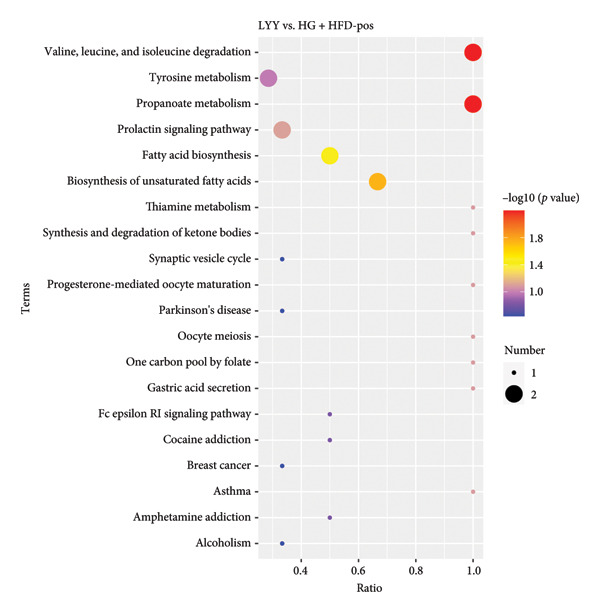
(a)

**Figure:**
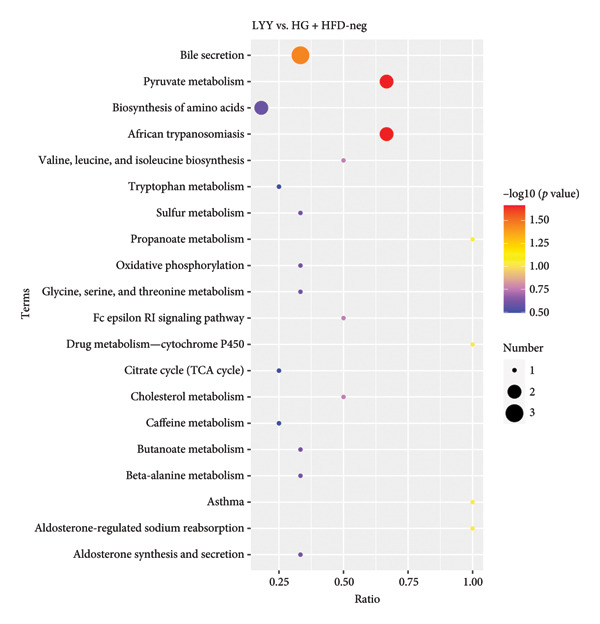
(b)

### 4.3. Effects of LYY on Gut Microbiota in Rats With T2DM

The dilution and hierarchical clustering curves from 16S rDNA sequencing showed gradual flattening, indicating an adequate amount of sequencing data with consistent sampling. This suggests that the data are sufficient and evenly distributed for reliable analysis (Figures 6(a) and 6(b)).

Figure 6(a) Observed features: The horizontal axis represents the amount of sequencing data, and the vertical axis represents the corresponding alpha diversity index. When the curve tends to be flat, it shows that the amount of sequencing data is gradual and reasonable, and more data will not have a significant impact on the alpha diversity index. Control (regular diet), HG + HFD (T2DM model, fed with high‐sugar and high‐fat diet), MET (positive metformin), and LYY (leaf ethanol extract of *D. longan Lour*.). Number of rats in each group = 6. (b) Relative abundance, in the horizontal direction, the richness of species is reflected by the width of the curve, and the higher the richness of species, the greater the span of the curve on the horizontal axis; in the vertical direction, the smoothness of the curve reflects the uniformity of species in the sample. The flatter the curve, the more uniform the species distribution is. Control (regular diet), HG + HFD (T2DM model, fed with high‐sugar and high‐fat diet), MET (positive metformin), and LYY (leaf ethanol extract of *D. longan Lour*.). Number of rats in each group = 6. (c) OTUs (operational taxonomic unit), in the figure, each circle represents a group, and the numbers in the overlapping parts of circles and circles represent the number of characteristic sequences shared among the groups, and the numbers without overlapping parts represent the number of characteristic sequences unique to the groups. Control (regular diet), HG + HFD (T2DM model, fed with high‐sugar and high‐fat diet), MET (positive metformin), and LYY (leaf ethanol extract of *D. longan Lour*.). Number of rats in each group = 6. (d) Chao 1, was used to evaluate the species richness of the gut microbiota. The horizontal axis represents the grouping, and the vertical axis represents the corresponding alpha diversity index values. Statistical significance by *T*‐test, Wilcoxon rank sum test, and Tukey test (Tukey and Kruskal–Wallis rank sum tests for grouping greater than 2). Control (regular diet), HG + HFD (T2DM model, fed with high‐sugar and high‐fat diet), MET (positive metformin), and LYY (leaf ethanol extract of *D. longan Lour*.). Number of rats in each group = 6. (e) Shannon was employed to measure gut microbiota diversity. The horizontal axis represents the grouping, and the vertical axis represents the corresponding alpha diversity index values. Statistical significance by *T*‐test, Wilcoxon rank sum test, and Tukey test (Tukey and Kruskal–Wallis rank sum tests for grouping greater than 2). Control (regular diet), HG + HFD (T2DM model, fed with high‐sugar and high‐fat diet), MET (positive metformin), and LYY (leaf ethanol extract of *D. longan Lour*.). Number of rats in each group = 6. (f) Simpson was employed to measure gut microbiota diversity. The horizontal axis represents the grouping, and the vertical axis represents the corresponding alpha diversity index values. Statistical significance by *T*‐test, Wilcoxon rank sum test, and Tukey test (Tukey and Kruskal–Wallis rank sum tests for grouping greater than 2). Control (regular diet), HG + HFD (T2DM model, fed with high‐sugar and high‐fat diet), MET (positive metformin), and LYY (leaf ethanol extract of *D. longan Lour*.). Number of rats in each group = 6.

**Figure:**
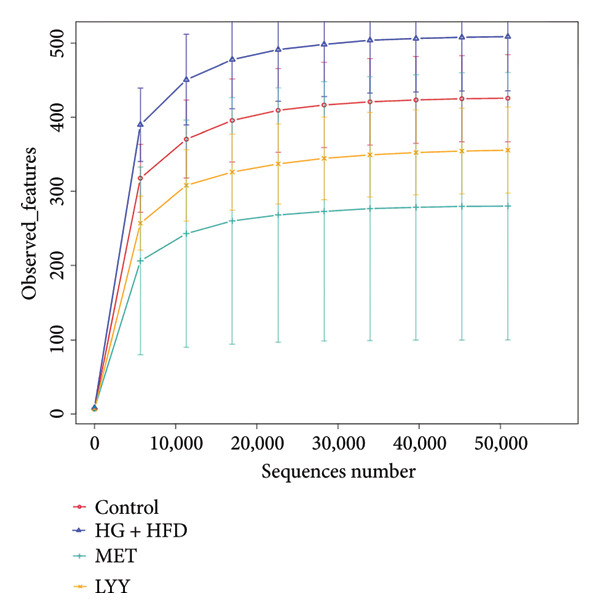
(a)

**Figure:**
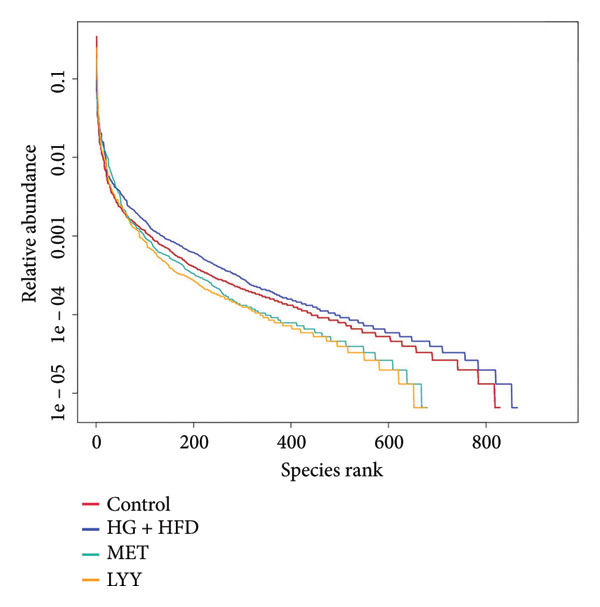
(b)

**Figure:**
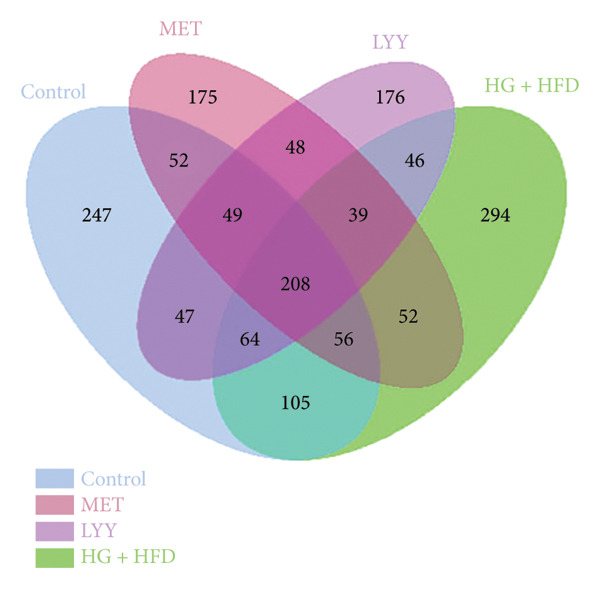
(c)

**Figure:**
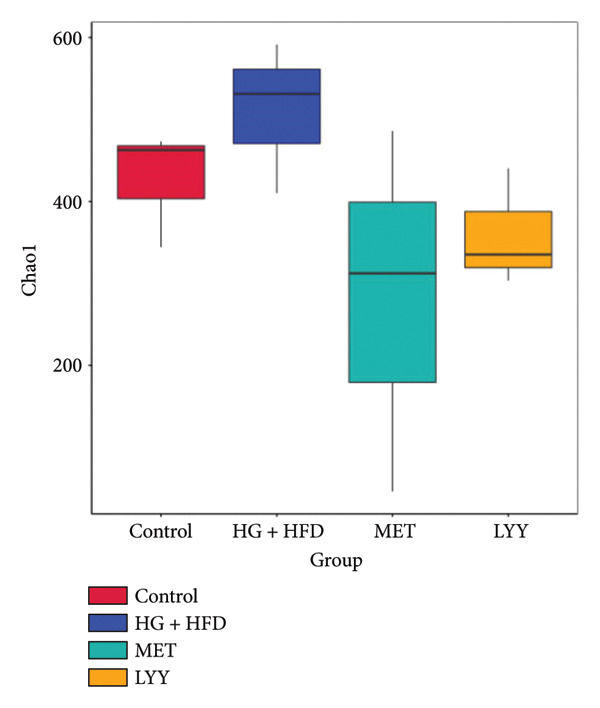
(d)

**Figure:**
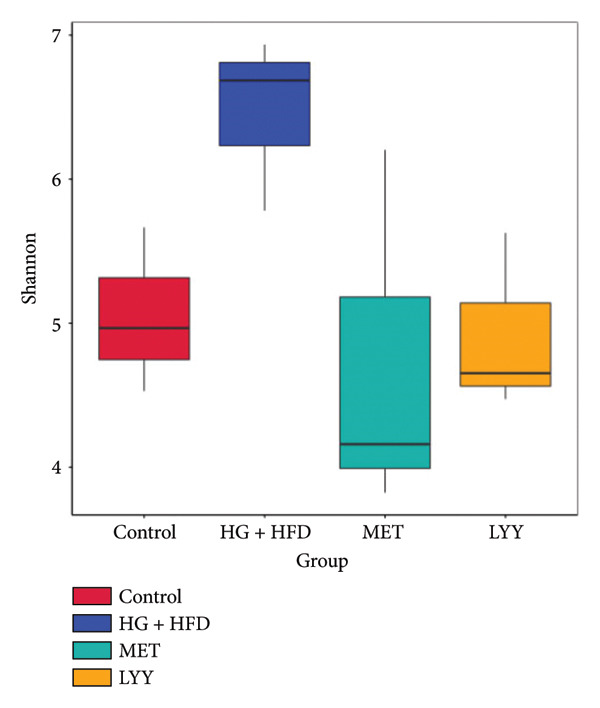
(e)

**Figure:**
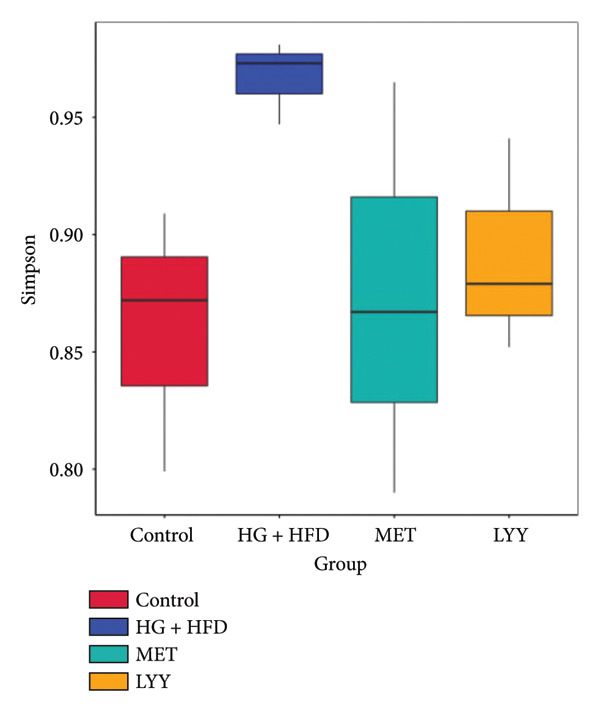
(f)

#### 4.3.1. α‐Diversity Analysis

The 16S rDNA sequence clustering showed 828, 864, 677, and 679 OTUs (Figure 6(c)) in the control, HG + HFD, MET, and LYY groups, respectively, with 208 common OTUs among the 4 groups. The Chao1 index (Figure 6(d)) was used to evaluate the species richness of the gut microbiota, whereas the Shannon (Figure 6(e)) and Simpson (Figure 6(f)) indices were employed to measure gut microbiota diversity. The HG + HFD group exhibited significantly elevated Chao1, Shannon, and Simpson indices compared with the control group, indicating a substantial increase in species richness and diversity in rats with T2DM. Following MET and LYY interventions, the OTU counts, species abundance, and gut microbiota diversity in rats with T2DM decreased, suggesting that LYY helps regulate the OUT count, abundance, and diversity of gut microbiota in rats with T2DM.

#### 4.3.2. β‐Diversity Analysis

Results of β‐diversity analysis are shown in Figure 7. In the weighted single‐segment distance model, principal coordinate analysis demonstrated clear separation of the HG + HFD group from the control, MET, and LYY groups. The LYY and control groups showed similar clustering, whereas the MET group showed significantly distinct clustering. In the unweighted single‐segment distance model (Figure 7(a)), the HG + HFD group was more easily distinguishable from the other three groups. The LYY and MET groups showed clustering trends similar to that of the control group, with LYY samples displaying more uniform distribution. These results indicate that T2DM caused substantial changes in gut microbiota structure and LYY treatment appears to effectively modulate this structure, restoring it closer to the structure of healthy rats.

Figure 7P‐CoA (principal coordinate analysis) of beta diversity analysis. (a) Weighte Unifrac; (b) Unweighted Unifrac. The abscissa represents one principal component, the ordinate represents the other principal component, and the percentage represents the contribution value of the principal component to the sample difference; each dot in the figure represents a sample, and the samples of the same group are represented using the same color. Control (regular diet), HG + HFD (T2DM model, fed with high‐sugar and high‐fat diet), MET (positive metformin), and LYY (leaf ethanol extract of *D. longan Lour*.). Number of rats in each group = 6.

**Figure:**
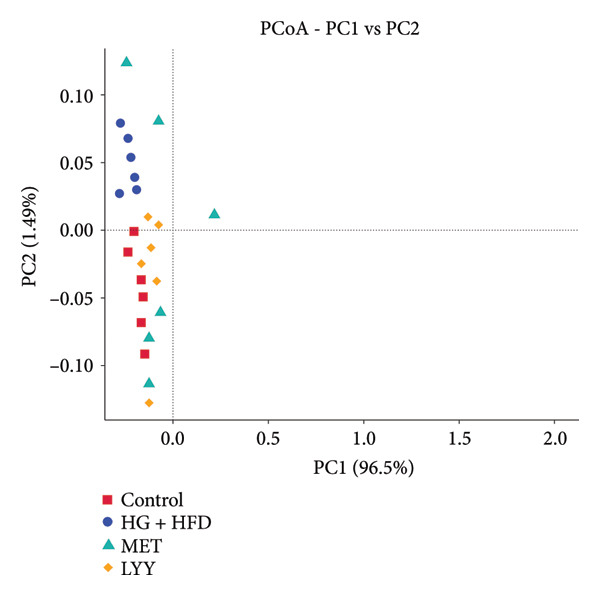
(a)

**Figure:**
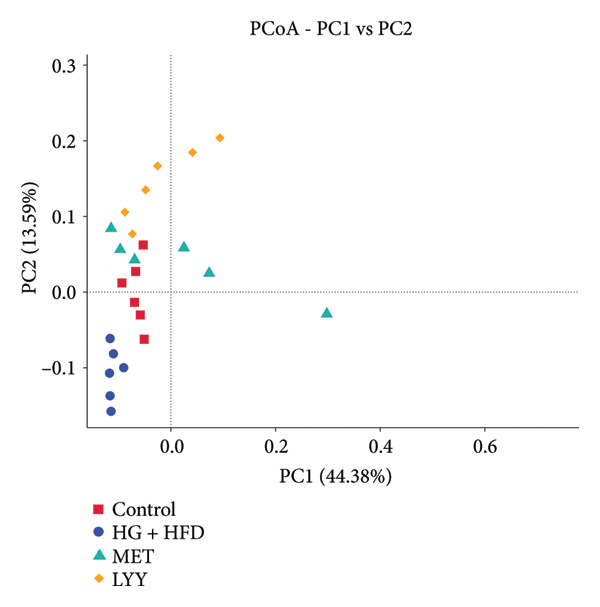
(b)

#### 4.3.3. Species Structure Analysis

**Figure 8:**
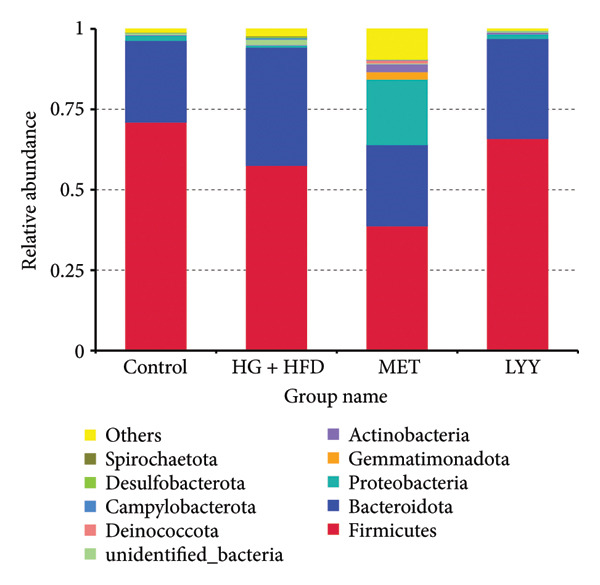
Species composition of intestinal flora at the phylum level in different groups of rats. Relative abundance of species at the level of phylum. The abscissa is the group name; the ordinate represents the relative abundance; others represent the sum of the relative abundances of all other phyla except these 10 phyla in the figure. Control (regular diet), HG + HFD (T2DM model, fed with high‐sugar and high‐fat diet), MET (positive metformin), and LYY (leaf ethanol extract of *D. longan Lour*.). Number of rats in each group = 6.

At the phylum level, the primary gut microbiota of rats across groups included *Firmicutes*, *Bacteroidota*, *Proteobacteria*, *Desulfobacterota*, *Gemmatimonadota*, *Actinobacteriota*, and *Deinococcota*, with *Firmicutes* and *Bacteroidota* being the dominant phyla and constituting > 55% of total relative abundance (Figure 8). Compared with the control group, the abundance of Firmicutes and *Proteobacteria* reduced by 13.5% and 2.0% in the HG + HFD group, whereas the abundance of *Bacteroidota* and *Desulfobacterota* increased by 11.3% and 1.0%, respectively. Compared with the HG + HFD group, the relative abundance of *Firmicutes* and *Bacteroidota* in the MET group decreased by 18.7% and 12.0%, whereas that of *Proteobacteria*, *Gemmatimonadota*, *Actinobacteriota*, and *Deinococcota* increased by 19.7%, 2.3%, 2.3%, and 1.0%, respectively. In the LYY group, the relative abundance of *Bacteroidota* decreased by 5.0%, whereas the abundance of *Firmicutes*, *Proteobacteria*, *Desulfobacterota*, and *Actinobacteriota* increased by 6.0%, 2.0%, 1.0%, and 1.0%, respectively.

**Figure 9:**
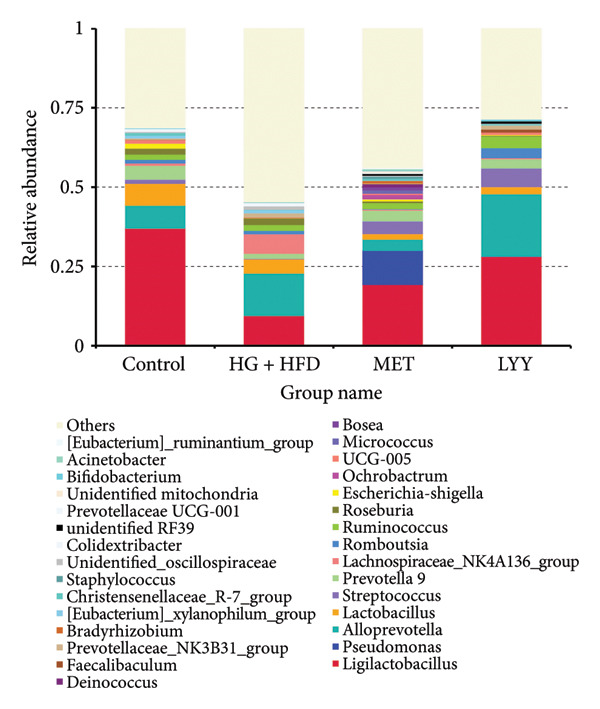
Species composition of intestinal flora at the genus level in different groups of rats. Relative abundance of species at the level of genus. The abscissa is the group name; the ordinate represents the relative abundance; others represent the sum of the relative abundances of all other phyla except these 30 genera in the figure. Control (regular diet), HG + HFD (T2DM model, fed with high‐sugar and high‐fat diet), MET (positive metformin), and LYY (leaf ethanol extract of *D. longan Lour*.). Number of rats in each group = 6.

At the genus level (Figure 9), the predominant genera of gut microbiota included *Ligilactobacillus*, *Alloprevotella*, *Pseudomonas*, *Lactobacillus*, *Prevotella 9*, *Streptococcus*, *Lachnospiraceae NK4A136 group*, *Romboutsia*, and *Ruminococcus*. Compared with the control group, the HG + HFD group showed reductions of 28.0%, 2.3%, and 2.8% in the relative abundance of *Ligilactobacillus*, *Lactobacillus*, and *Prevotella 9* as well as increases of 6.1% and 5.4% in the relative abundance of *Alloprevotella* and *Lachnospiraceae NK4A136 group*, respectively. Compared with the HG + HFD group, the MET group showed a decrease in the abundance of *Alloprevotella*, *Lactobacillus*, and *Lachnospiraceae NK4A136 group* by 9.8%, 2.8%, and 5.8%, and an increase in the abundance of *Ligilactobacillus*, *Pseudomonas*, *Streptococcus*, and *Prevotella 9* by 9.8%, 10.7%, 3.9%, and 1.8%, respectively. In the LYY group, *Lactobacillus* abundance decreased by 2.1%, whereas the abundance of *Ligilactobacillus*, *Alloprevotella*, *Streptococcus*, *Prevotella 9*, *Romboutsia*, and *Ruminococcus* increased by 18.6%, 6.3%, 5.7%, 1.3%, 2.1%, and 1.8%, respectively. These findings suggest that LYY intervention modulates the relative abundance of various gut microbiota in rats with T2DM.

#### 4.3.4. Discriminant Analysis of Species Differences

The MetaStat method was used to validate population abundance data, obtaining *p*‐values between groups and identifying species with significant variances. Compared with the control group, the HG + HFD group showed an increase in the abundance of six bacterial genera, such as *Desulfobacterota*, *Lachnospiraceae NK4A136 group*, and *Turicibacter*, whereas the abundance of *Ligilactobacillus* decreased. In the MET group, the abundance of *Firmicutes*, *Desulfobacterota*, *Elusimicrobia*, *Lachnospiraceae NK4A136 group*, *Romboutsia*, and *Turicibacter* decreased compared with the HG + HFD group, whereas the abundance of *Ligilactobacillus*, *Streptococcus*, and *Christensenellaceae R-7 group* increased significantly. The LYY group exhibited significant decreases in the abundance of *Desulfobacterota*, *Spirochaetota*, *Elusimicrobia*, *Lachnospiraceae NK4A136 group*, *Roseburia*, *Colidextribacter*, and *Oscillibacter* compared with the HG + HFD group (*p* < 0.05).

### 4.4. Association Analysis of Metabolomics and Gut Microbiota in Rats With T2DM

**Figure 10:**
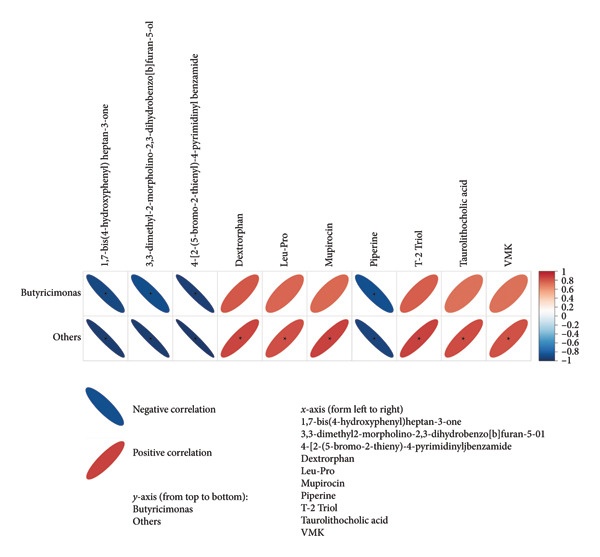
Heat map of the correlation between fecal metabolites and *Butyricimonas*. Positive ion model. The horizontal direction of the figure shows the differential metabolites, and the vertical direction shows the differential bacteria. The legend on the right is the correlation coefficient; the redder the color, the stronger the positive correlation; the bluer the color, the stronger the negative correlation; and the flatter the ellipse, the higher the absolute value of the correlation. In the result figure, the asterisk (^∗^) marked part is *p* ≤ 0.05. Statistical significance by the Pearson statistical method.

**Figure 11:**
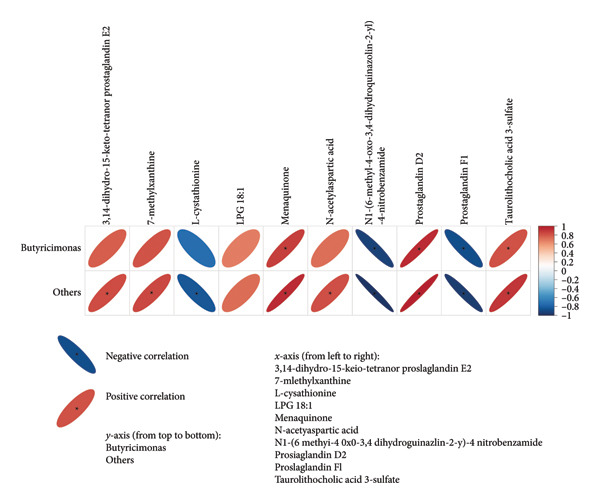
Heat map of the correlation between fecal metabolites and *Butyricimonas*. Negative ion model. The horizontal direction of the figure shows the differential metabolites, and the vertical direction shows the differential bacteria. The legend on the right is the correlation coefficient; the redder the color, the stronger the positive correlation; the bluer the color, the stronger the negative correlation; and the flatter the ellipse, the higher the absolute value of the correlation. In the result figure, the asterisk (^∗^) marked part is *p* ≤ 0.05. Statistical significance by the Pearson statistical method.

**Figure 12:**
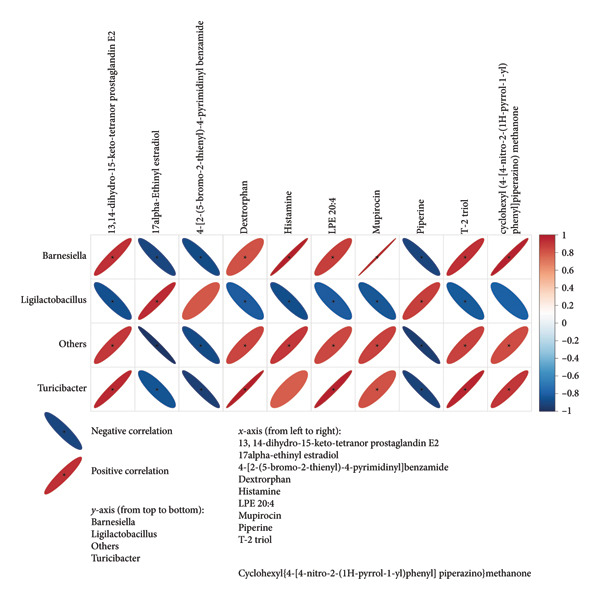
Heat map of the correlation between fecal metabolites and *Ligilactobacillus* and *Turicimonas*. Positive ion model. The horizontal direction of the figure shows the differential metabolites, and the vertical direction shows the differential bacteria. The legend on the right is the correlation coefficient; the redder the color, the stronger the positive correlation; the bluer the color, the stronger the negative correlation; and the flatter the ellipse, the higher the absolute value of the correlation. In the result figure, the asterisk (^∗^) marked part is *p* ≤ 0.05. Statistical significance by the Pearson statistical method.

**Figure 13:**
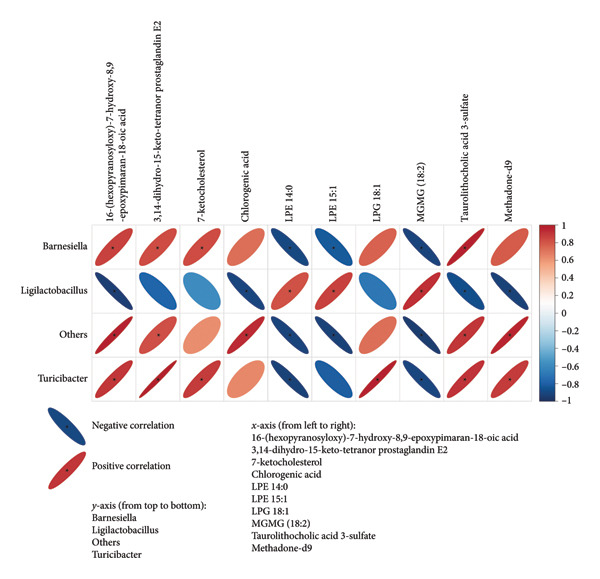
Heat map of the correlation between fecal metabolites and *Ligilactobacillus* and *Turicimonas*. Negative ion model. The horizontal direction of the figure shows the differential metabolites, and the vertical direction shows the differential bacteria. The legend on the right is the correlation coefficient; the redder the color, the stronger the positive correlation; the bluer the color, the stronger the negative correlation; and the flatter the ellipse, the higher the absolute value of the correlation. In the result figure, the asterisk (^∗^) marked part is *p* ≤ 0.05. Statistical significance by the Pearson statistical method.

**Figure 14:**
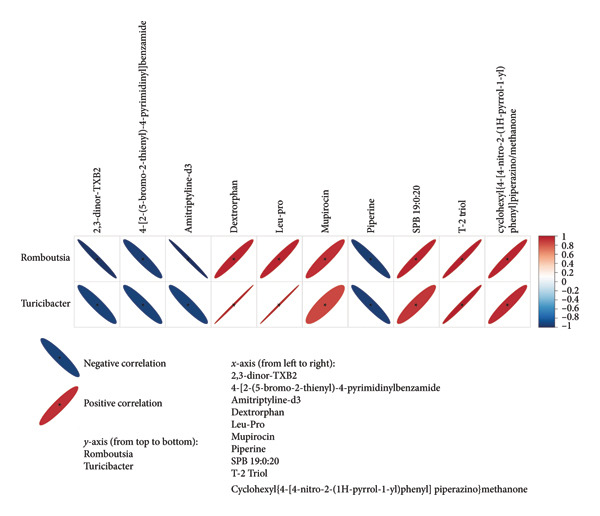
Heat map of the correlation between fecal metabolites and *Romboutsia* and *Turicimonas*. Positive ion model. The horizontal direction of the figure shows the differential metabolites, and the vertical direction shows the differential bacteria. The legend on the right is the correlation coefficient; the redder the color, the stronger the positive correlation; the bluer the color, the stronger the negative correlation; and the flatter the ellipse, the higher the absolute value of the correlation. In the result figure, the asterisk (^∗^) marked part is *p* ≤ 0.05. Statistical significance by the Pearson statistical method.

**Figure 15:**
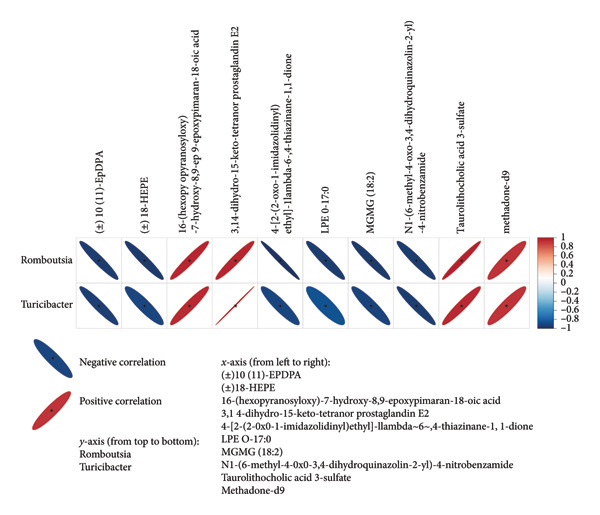
Heat map of the correlation between fecal metabolites and *Romboutsia* and *Turicimonas*. Negative ion model. The horizontal direction of the figure shows the differential metabolites, and the vertical direction shows the differential bacteria. The legend on the right is the correlation coefficient; the redder the color, the stronger the positive correlation; the bluer the color, the stronger the negative correlation; and the flatter the ellipse, the higher the absolute value of the correlation. In the result figure, the asterisk (^∗^) marked part is *p* ≤ 0.05. Statistical significance by the Pearson statistical method.

To explore the link between metabolomics and gut microbiota, 61 differential metabolites were correlated with gut microbiota, such as *Desulfobacterota*, *Firmicutes*, *Lachnospiraceae NK4A136 group*, *Turicibacter*, *Ligilactobacillus*, and *Romboutsia*. Spearman correlation analysis revealed significant associations between certain metabolites and gut microbiota. For example, *Butyricimonas* was positively correlated with taurolithocholic acid 3‐sulfate and negatively correlated with piperine and 4‐[2‐(5‐bromo‐2‐thienyl)‐4‐pyrimidinyl]benzamide (Figures 10 and 11). *Turicibacter* was positively correlated with metabolites, such as dextrorphan, T‐2 triol, cyclohexyl {4‐[4‐nitro‐2‐(1H‐pyrrol‐1‐yl) phenyl] piperazino} methanone, 13,14‐dihydro‐15‐keto‐tetranor prostaglandin E2, 7‐ketocholesterol, LPG 18: 1, taurolithocholic acid 3‐sulfate, Leu‐Pro, 16‐(hexopyranosyloxy)‐7‐hydroxy‐8, and 9‐epoxypimaran‐18‐oic acid (Figures 12, 13, 14, 15) but negatively correlated with 4‐[2‐(5‐bromo‐2‐thienyl)‐4‐pyrimidinyl]benzamide and piperine (Figure 12). *Ligilactobacillus* was positively correlated with piperine and 17‐alpha‐ethinyl estradiol and negatively correlated with 13,14‐dihydro‐15‐keto‐tetranor prostaglandin E2, dextrorphan, histamine, mupirocin, T‐2 triol, 16‐(hexopyranosyloxy)‐7‐hydroxy‐8, 9‐epoxypimaran‐18‐oic acid, and taurocholic acid 3‐sulfate (Figures 12 and 13). Similarly, *Romboutsia* was positively correlated with dextrorphan, Leu‐Pro, mupirocin, T‐2 triol, cyclohexyl {4‐[4‐nitro‐2‐(1H‐pyrrol‐1‐yl) phenyl] piperazino} methanone, and taurolithocholic acid 3‐sulfate but negatively correlated with 4‐[2‐(5‐bromo‐2‐thienyl)‐4‐pyrimidinyl] benzamide and piperine (Figures 14 and 15).

## 5. Discussion

In this chapter, a rat model of T2DM is established to evaluate the efficacy of LYY in treating T2DM from body weight, FBG, blood lipid, and other indicators. Because the STZ model method of the T2DM model has been maturely applied, the cost of this model is lower than that of the spontaneous diabetes model [15]. However, this method has certain limitations: The body weight of rats will be affected by STZ, possibly due to the limitations of STZ, which can lead to decreased insulin secretion and weight loss [20], and it will affect the judgment of weight results. So, it would be better if the T2DM model was induced by feeding a high‐sugar and high‐fat diet, and the compared rats were fed a high‐sugar and high‐fat diet with common feed in the LYY group.

Fecal metabolites are products of complex metabolic activities of gut microbiota, and high‐throughput metabolomics screening offers valuable insights into the underlying pathophysiology of diabetes [21]. The regulatory pathways of fecal metabolites in T2DM primarily involve the metabolism of valine, leucine, and isoleucine; biosynthesis of fatty acids and unsaturated fatty acids; metabolism of propionic acid and pyruvate; and bile secretion. Valine, leucine, and isoleucine are classified as branched‐chain amino acids because of their branched chains and common structural characteristics [22]. Nie indicated that branched‐chain amino acids are mainly involved in glucose, lipid, and protein metabolism [23], and elevated levels of these amino acids are closely linked to insulin resistance in T2DM [24–28]. Metabolomics analysis in this study revealed that leucine was enriched in rats with T2DM, and branched‐chain amino acid levels were significantly downregulated after LYY treatment.

Unsaturated fatty acids, which play a critical role in lowering cholesterol and TG content, are also closely associated with T2DM [29–31]. Following LYY intervention, metabolites, such as 5‐OxoETE, LPE 15:0, and LPS 13:0, which are all unsaturated fatty acids, were upregulated, consistent with observed reductions in TC and TG content.

Propionic acid, a major short‐chain fatty acid (SCFA), promotes glucose tolerance [32] and enhances insulin sensitivity, thereby regulating blood sugar fluctuations [33]. Additionally, pyruvate, the final product of glycolysis, is a key compound facilitating the conversion of sugars, fats, and amino acids through the catabolic pathway involving acetyl‐CoA and tricarboxylic acid, thereby influencing metabolic homeostasis involving three major nutrients [34]. In this study, LYY intervention in rats with T2DM led to increased levels of 2‐isopropylmalic acid, a pyruvate derivative, and it is suggested that LYY can regulate pyruvate metabolism and may play a therapeutic role in T2DM associated with interfering with glycolysis and gluconeogenesis pathways.

Bile acids, the final products in cholesterol metabolism [35], play a crucial role in regulating glucose and lipid metabolism. Through pathways involving nuclear receptors, such as the nitric alcohol X receptor, and membrane receptors, such as G protein–coupled bile acid receptor 5, bile acids stimulate the secretion of glucagon‐like peptide‐1. This cascade helps improve insulin sensitivity, enhances insulin responsiveness, and supports the overall regulation of glucose and lipid metabolism [36]. Studies have consistently shown that bile acid level plays an important role in the development of T2DM; for example, insulin resistance is positively correlated with high bile acid levels in diabetic patients [37–41]. Following LYY intervention, T2DM rats showed decreased levels of taurolithocholic acid 3‐sulfate, indicating that LYY could regulate bile acid metabolism and thus glucose metabolism in T2DM.

Gut microbiota, comprising over a thousand bacterial species [42], markedly influences T2DM development through effects on glucose tolerance and insulin resistance [43]. Alterations in microbial diversity, abundance, and structure, along with related metabolite changes, play crucial roles in T2DM progression [44, 45]. Previous studies have shown that individuals with diabetes exhibit a substantial decrease in *Bifidobacteria*, *Clostridium*, and *Firmicutes* in their gut microbiota, with a marked increase in *β- Proteobacteria* [46, 47]. Impaired glucose tolerance is a common symptom of diabetes; if not properly managed, it can exacerbate the condition and jeopardize patient health. Gut microbiota plays a crucial role in the progression of impaired glucose tolerance [48]. For instance, *Lactobacillus gasseri* in the upper small intestine is suppressed by a high‐fat diet, which can impair glucose tolerance. Conversely, the activation of fatty acids promotes the involvement of *L. gasseri* in the synergistic regulation of small intestinal glucose, effectively improving glucose tolerance [49, 50]. This suggests that gut microbiota interferes with glucose tolerance and indirectly regulates glucose metabolism balance [51].

In this study, rats with T2DM showed reduced abundance of *Firmicutes* and increased abundance of *Bacteroides*, with a significant change in the α‐diversity index, supporting the association between T2DM and dysregulation of gut microbiota. Firmicutes play a role in insulin signal transduction, glucose metabolism, and improved insulin resistance in T2DM [52], and the *Firmicutes/Bacteroides* ratio is considered a marker for diet‐induced gut microbiota dysbiosis. Following LYY intervention, the abundance of *Firmicutes* and *Bacteroides* normalized, the stability of bacteria and bacilli improved, the symptoms of diet‐induced gut microbiota disorder were suppressed, and the structural homeostasis of gut microbiota was restored. Additionally, the α‐diversity index shifted closer to that of normal rats, demonstrating LYY’s potential to restore microbial balance in rats with T2DM.

At the genus level, the increased abundance of *Turicibacter*, a pathogenic bacterium enriched in the HG + HFD group, is a hallmark of structural dysbiosis in gut microbiota [53]. LYY intervention increased the abundance of beneficial bacteria, such as *Ligilactobacillus* and *Christensenellaceae R-7 group*, and reduced the abundance of *Desulfobacterota*, *Spirochaetota*, *Elusimicrobia*, *Roseburia*, *Colidextribacter*, and *Oscillibacter*. *Ligilactobacillus* and *Christensenellaceae R-7 group* are associated with glucose and lipid metabolism. *Lactobacillus*, an important probiotic in the human gut, plays a beneficial role in reducing oxidative stress [54]. *Christensenellaceae R-7 group*, which is also a probiotic in the gut and mucosa of humans and animals, reduces proinflammatory cytokine levels and improves intestinal mucosal barrier function [55]. Meanwhile, *Ligilactobacillus* could increase the concentrations of acetic acid, propionic acid, butyric acid, and valeric acid, indicating that *Ligilactobacillus* could promote the production of SCFAs [56]. Both *Christensenellaceae* strains produce acetic acid and butyric acid [57]. Following LYY intervention, a decline in the relative abundance of *Desulfobacterota*, *Colidextribacter*, and other bacteria was observed, aligning with previous findings [58, 59].

In the association analysis of metabolomics and gut microbiota, a variety of fecal differential metabolites are related to the microbiota, suggesting that the occurrence of T2DM can cause changes in gut microbiota communities, and then affect metabolic abnormalities. In metabolomics studies, we found that T2DM caused high bile acid levels in rats, and bile acids were closely related to gut microbiota. Gut microbiota can regulate the key enzymes of bile acid metabolism to promote the anabolic metabolism or biotransformation of bile acids, and also produce secondary bile acids as G protein–coupled bile acid receptors, which regulate bile acid metabolism with nuclear receptor FXR, further stimulate the release of pancreatic GLP‐1 and regulate glucose metabolism [60]. The results of our study showed that the metabolite taurolithocholic acid 3‐sulfate was significantly upregulated in gut microbiota, such as *Turicibacter* and *Romboutsia* in T2DM model rats. The association analysis results showed that *Turicibacter* and *Romboutsia* were positively correlated with taurolithocholic acid 3‐sulfate, indicating that the increase in the abundance of intestinal microorganisms *Turicibacter* and *Romboutsia* in T2DM rats inhibited bile acid metabolism. However, the abundance of *Ligilactobacillus* was upregulated, and the taurolithocholic acid 3‐sulfate level was downregulated after intervention in LYY. The association analysis showed that *Ligilactobacillus* was negatively correlated with taurolithocholic acid 3‐sulfate. To sum up, it is speculated that LYY can upregulate the abundance of *Ligilactobacillus*, and then, *Ligilactobacillus* promotes the taurolithocholic acid 3‐sulfate metabolism, stimulates the pancreas to release substances to promote pancreatic function, and finally regulates glucose metabolism to play a therapeutic role in T2DM.

Regarding the setting of the high‐fat diet control group (without STZ), we have considered the interaction between diet and STZ in the early stage of experimental design, but limited by factors, such as experimental period, animal ethics, and sample size, we did not set a separate high‐fat diet control group without STZ. We plan to add a high‐fat diet + no STZ group to the extension experiment and further distinguish the effects of the two factors by the following indicators: Glucose Metabolism Indicators: OGTT and insulin release test; Lipid Metabolism Indicators: free fatty acids and cholesterol subcomponents; Indicators of Inflammation and Oxidative Stress: TNF‐α and MDA.

As mentioned earlier, the previous research results of our research group showed that the main active ingredients of ethanol extract of *D. longan Lour.* leaves were quercetin, luteolin, kaempferol, and other flavonoids. Studies have shown that quercetin can significantly reduce FBG, TG, alanine aminotransferase, and aspartate aminotransferase levels in DM rats (*p* < 0.05), which significantly increased HDL levels (*p* < 0.05), and 16S rRNA results showed that quercetin could improve the abundance of intestinal flora of 9 genera including *Streptococcus* and *Butyricicoccus*. Untargeted metabolomics results revealed that quercetin regulates metabolic pathways, such as sphingolipid metabolism, glycerophospholipid metabolism, and steroid biosynthesis [61]. Kaempferol derivatives can increase the phosphorylation levels of *p*‐AKT (473) and its downstream GSK3β, thereby increasing glycogen synthesis and alleviating insulin resistance [62]. The results of this study showed that after LYY intervention, the levels of phospholipids, such as LPE15: 0 and LPS13: 0, increased. It has also been shown that gallic acid can reduce liver TG levels and serum ALT, AST, TC, and TG levels [63]. Therefore, we speculate that these compounds in LYY are involved in the pathways that regulate the metabolic level and gut microbiota abundance in T2DM rats and play an important role.

However, due to limitations, such as the conditions of the detection platform during the experimental period, we were unable to directly determine SCFAs, which is one of the limitations of this study. Nevertheless, we indirectly supported the association between microbiota function and metabolic improvement through the following analyses: The microbiota with significantly increased abundance in this study (such as *Ligilactobacillus* and *Christensenellaceae*) has been confirmed by multiple studies as the main producers of SCFAs [56, 57]. Changes in their abundance can indirectly reflect alterations in SCFA synthesis capacity. Predictive analysis of microbiota functional genes showed that the abundance of metabolites related to SCFAs (such as the pyruvate derivative 2‐isopropylmalic acid) was significantly upregulated in the LYY group (*p* < 0.05), suggesting a potential trend of changes in the metabolic function of the microbiota. For subsequent studies, we plan to include direct verification of the “microbiota‐SCFAs‐metabolic improvement” axis. Specifically, we will conduct fecal microbiota transplantation combined with SCFA intervention experiments to further clarify the functional mechanism. Additionally, we will directly detect the SCFA production rate of *Ligilactobacillus* strains through in vitro culture or verify the direct impact of *Christensenellaceae* on intestinal SCFA metabolism through experiments, such as fecal microbiota transplantation and colonization in germ‐free animals to clarify their functional contributions.

In addition, this study established a T2DM model in rats for experiments. The intestinal length, digestive cycle, immune status, and living environment (laboratory SPF conditions vs. human natural exposure environment) of rodents may lead to differences in the colonization patterns and functional performance of the microbiota compared to humans, and these variables will have a certain impact on the research results. For example, there are significant differences between rodents and humans in terms of dominant intestinal phyla (such as the ratio of *Bacteroidetes* to *Firmicutes*), core species (such as the difference between *Lactobacillus* in mice and *Bacteroides* in humans), and metabolites (such as the types and abundance of SCFAs). These differences are related to factors, such as differences in dietary sources, feeding patterns, large intestine length, living environment, and biological rhythms between rats and humans, which may directly affect the extrapolation of experimental conclusions [64, 65]. This is another limitation of this study. But this kind of difference does not deny the value of animal models, but by facing up to their limitations, it demarcates the boundary for the interpretation of research results and at the same time provides direction for subsequent cross‐species verification—this is precisely the key to improving the rigor and influence of research core logic. To improve the understanding of the mechanism of LYY in treating T2DM, we will further verify it in the future through methods, such as organoid models, nonhuman primate models, or clinical cohort correlation analysis.

## 6. Conclusion

This study integrated metabolomics and gut microbiota analysis to explore the mechanism by which LYY treatment benefits rats with T2DM. The findings suggest that LYY effectively alleviates T2DM symptoms associated with several pathways: valine, leucine, and isoleucine metabolism; fatty acid and unsaturated fatty acid biosynthesis; propionic acid, pyruvate, and bile acid metabolism; and upregulation of *Ligilactobacillus* and *Christensenellaceae R-7 group* and downregulation of *Desulfobacterota*, *Colidextribacter*, and *Oscillibacter*. This modulation interferes with the impaired pancreas and indirectly regulates glucose metabolism balance.

## Consent

The authors have nothing to report.

## Disclosure

All authors have read and agreed to the published version of the final manuscript.

## Conflicts of Interest

The authors declare no conflicts of interest.

## Author Contributions

Chunlian Lu contributed to the conceptualization and writing. Piaoxue Zheng contributed to the conceptualization. Sisi Chen contributed to the visualization. Yanli Liang and Yuxin Jiang contributed to the data curation. Anqi Huo and Jingjing Xie contributed to the formal analysis. Jiawen Peng contributed to the investigation. Yuming Ma contributed to the methodology. Jiali Wei and Zhidong Ma contributed to the writing–original draft. Hua Zhu and Jie Liang contributed to the writing–review. Chunlian Lu and Piaoxue Zheng contributed equally to the work.

## Funding

This work was supported by the National Natural Science Foundation of China (No. 82160771), NATCM’s Project of High‐Level Construction of Key TCM Disciplines: Traditional Medicine of Chinese Minority (Zhuang Medicine) (No. zyyzdxk‐2023165), Huang Danian Style Teacher Team From Universities in Guangxi Zhuang Autonomous Region “Traditional Chinese Medicine Inheritance and Innovation Teacher Team” (No. [2023]31), Guangxi One Thousand Young and Middle‐Aged College and University Backbones Teachers Cultivation Program (No. (2019)5), Guangxi Traditional Chinese Medicine Multidisciplinary Cross Innovation Team Project (No. GZKJ2309), Guangxi Key Discipline Of Traditional Chinese Medicine Zhuang Pharmacy (No. GZXK‐Z‐20‐64), The First‐Class Subject of Traditional Chinese Medicine (Ethnic Pharmacy) in Guangxi (No. (2018)12), Guangxi Autonomous Region Level College Students Innovation Training Program Project (No. S202410600067), Research and Training Project for College Students at Guangxi University of Chinese Medicine (No. 2024DXS16), and the third batch of Cultivating High‐level Talent Teams in the “Qi Huang Project” of the Guangxi University of Chinese Medicine (No. 202406).

## Data Availability

The data that support the findings of this study are available from the corresponding author upon reasonable request.
